# The Protective Effects of *Lactobacillus reuteri* Combined With *Clostridium butyricum* Miyairi 588 on Intestinal Barrier Function, Water Transport, and Oxidative Stress in a Rat Model of 5FU‐Induced Diarrhea

**DOI:** 10.1002/fsn3.70318

**Published:** 2025-05-23

**Authors:** Sheau‐Chung Tang, Wen‐Chen Chuang, Jiuan‐Miaw Liao, Yi‐Hsuan Tsai, Liang‐Chuan Chen, Jiunn‐Wang Liao, Jiunn‐Liang Ko, Chu‐Chyn Ou

**Affiliations:** ^1^ Department of Nursing National Taichung University of Science and Technology Taichung Taiwan; ^2^ School of Medicine Chung Shan Medical University Taichung Taiwan; ^3^ Department of Physiology, School of Medicine Chung Shan Medical University Taichung Taiwan; ^4^ Department of Nutrition Chung Shan Medical University Taichung Taiwan; ^5^ Department of Food Science and Biotechnology National Chung Hsing University Taichung Taiwan; ^6^ Graduate Institute of Veterinary Pathobiology National Chung Hsing University Taichung Taiwan; ^7^ Institute of Medicine Chung Shan Medical University Taichung Taiwan; ^8^ Department of Medical Oncology and Chest Medicine Chung Shan Medical University Hospital Taichung Taiwan; ^9^ Department of Nutrition Chung Shan Medical University Hospital Taichung Taiwan

## Abstract

5‐Fluorouracil (5FU) is a commonly employed and highly effective chemotherapeutic agent in clinical oncology. Nevertheless, one of the most frequent and debilitating adverse effects associated with 5FU treatment is diarrhea. These gastrointestinal complications can affect patients' quality of life and adherence to treatment regimens. Consequently, addressing and mitigating diarrhea during 5FU therapy presents a critical and urgent challenge in oncological care. This study investigated whether probiotic *Lactobacillus reuteri* combined with *Clostridium butyricum* Miyairi 588 (LCs) can alleviate 5FU‐induced diarrhea and the potential mechanism. Wistar rats received 5FU (50 mg/kg, intraperitoneal injection) for 5 consecutive days to establish a 5FU‐induced colitis diarrhea model. LCs were administered 15 days before the 5FU injection and continued until the day of sacrifice. Tissue morphology, inflammatory and oxidative stress markers, as well as the expression of mRNA related to intestinal barrier integrity, apoptosis, and aquaporins (AQPs) were evaluated in the colon tissue samples. These analyses used hematoxylin and eosin staining, enzyme‐linked immunosorbent assay (ELISA), and quantitative real‐time reverse transcription‐polymerase chain reaction (qRT‐PCR) techniques. Additionally, the concentrations of short‐chain fatty acids (SCFAs) were measured using gas chromatography‐flame ionization detection (GC‐FID) analysis. In this colitis model, LCs mitigated 5FU‐induced weight loss, diarrhea, bloody stool, shortened colon length, and colonic histopathology. Treatment with LCs resulted in reduced levels of MDA, TNF‐α, IL‐1β, and MPO activity, as well as decreased mRNA expression of IFN‐γ, AKT, NF‐κB, TNF‐α, and iNOS. Additionally, LCs significantly downregulated the expression of VCAM‐1, CXCL4, MAPK, and caspase‐3, while upregulating the tight junction protein occludin expression. LCs also notably diminished the mRNA expression levels of AQP7, VIP, and PKA. This study demonstrates that LCs have therapeutic effects on colitis, primarily through their antioxidant properties, anti‐apoptotic effects, mucosal barrier integrity maintenance, neutrophil infiltration reduction, and inflammatory cytokines and aquaporin expression modulation.

## Graphical abstract

I recommend using Figure Graphical abstracts for the graphical table of contents and publication cover. This figure effectively illustrates probiotics LCs have therapeutic effects in a rat model of 5FU‐induced diarrhea, making it a compelling visual summary of our research.

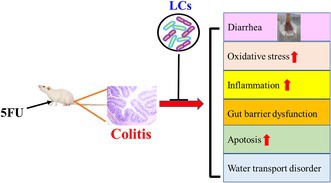

## 1 Introduction

Colitis is a well‐recognized adverse effect of 5‐fluorouracil (5FU) chemotherapy, clinically manifesting as weight loss, hematochezia, diarrhea, and reduced colon length. The incidence of 5FU‐induced diarrhea ranges from 40% to 70%, significantly impairing patients' quality of life. Severe diarrhea may lead to malnutrition, electrolyte imbalance, immunosuppression, and, in some cases, fatal outcomes (Siritientong et al. 2025; Yue et al. 2025). The pathogenesis of 5FU‐induced colitis is primarily driven by inflammation, oxidative stress, and apoptosis, with excessive activation of neutrophils and macrophages playing a key role in disease progression (Shen et al. 2021; Xiang et al. 2020). Although anti‐ulcer agents and antibiotics have shown partial efficacy in alleviating 5FU‐induced intestinal mucositis, their clinical application remains limited (Chen et al. 2022). Recent studies suggest that probiotics may help manage chemotherapy‐induced diarrhea and mitigate intestinal mucositis in cancer patients undergoing 5FU‐based regimens (Siritientong et al. 2025).

Probiotics are generally considered safe and well‐tolerated, and are increasingly used as adjunctive therapies during radiotherapy and chemotherapy (Chrysostomou et al. 2023; Wang et al. 2023). Their health benefits have been linked to various mechanisms, including antioxidant activity, immune modulation, and intestinal barrier preservation (Maftei et al. 2024). Both clinical and preclinical evidence suggest that probiotics can alleviate gastrointestinal disorders, such as diarrhea, abdominal discomfort, and inflammatory bowel disease, by modulating oxidative stress and attenuating inflammatory responses (Ariyoshi et al. 2021; Huang et al. 2023). However, the biological effects of probiotics are strain‐specific, and their therapeutic efficacy varies accordingly. Despite this variability, targeting gut microbiota remains a promising strategy for the prevention and management of 5FU‐induced mucositis (Chen et al. 2022; Justino et al. 2020; Shen et al. 2021; Yeung et al. 2021). In addition to modulating inflammatory responses, probiotics may influence the expression of adhesion molecules such as intercellular adhesion molecule‐1 (ICAM‐1) and vascular cell adhesion molecule‐1 (VCAM‐1), both of which play crucial roles in leukocyte recruitment during inflammation (Li et al. 2017). Although probiotic‐mediated inhibition of adhesion molecule upregulation has been associated with symptom relief in colitis models, the underlying mechanisms remain poorly defined, and the results across studies are inconsistent (Chu et al. 2010; Rezazadeh et al. 2023).

The probiotic mixture LCs, composed of *L. reuteri* and *C. butyricum* Miyairi 588, has demonstrated potent anti‐inflammatory properties (Ariyoshi et al. 2021; Yue et al. 2025). *C. butyricum* has been reported to reduce serum pro‐inflammatory cytokines and enhance the expression of intestinal barrier proteins, including claudin‐3, occludin, zonula occludens‐1 (ZO‐1), and zonula occludens‐2 (ZO‐2), thereby reinforcing intestinal integrity (Yu et al. 2024). Additionally, *C. butyricum* promotes IL‐10 production in intestinal macrophages via a TLR2‐MyD88‐dependent mechanism, contributing to its protective effects against dextran sulfate sodium (DSS)‐induced colitis (Hagihara et al. 2020). Similarly, recent studies have shown that *L. reuteri* alleviates intestinal inflammation by modulating gut microbiota, enhancing SCFA production, and reinforcing epithelial barrier function. These effects are linked to its ability to suppress pro‐inflammatory cytokines, regulate immune responses, and secrete bioactive metabolites such as reuterin and organic acids (Lee et al. 2025; Yue et al. 2025).

Although previous studies have highlighted the therapeutic potential of probiotics in mitigating chemotherapy‐induced intestinal mucositis, the strain‐specific efficacy and the underlying mechanisms remain inconsistent and not fully elucidated (Lopez‐Gomez et al. 2023). Our unpublished data demonstrated that the combination of *L. reuteri* and *C. butyricum* (LCs) exhibited superior protective effects compared to single‐strain treatments in a cisplatin‐induced intestinal mucosal injury model. Based on this preliminary evidence, we hypothesized that this dual‐strain formulation could also exert protective effects against 5FU‐induced intestinal mucositis. Therefore, the present study aimed to investigate whether preadministration of LCs could alleviate 5FU‐associated diarrhea and colitis in a rat model by modulating inflammatory responses, oxidative stress, apoptosis, adhesion molecule expression, and intestinal barrier integrity. Our findings not only support the potential of LCs as a probiotic‐based intervention for chemotherapy‐induced mucosal injury but also underscore the significance of strain combinations and rational dosing strategies in enhancing the efficacy of probiotic therapies. These results provide novel insights into the design of strain‐combination approaches for probiotic interventions and suggest that LCs may represent a promising alternative for supportive care in managing chemotherapy‐induced gastrointestinal toxicity.

## 2 Materials and Methods

### 2.1 Animals

Adult 4‐week‐old Wistar rats (100‐125 g) were obtained from BioLASCO Taiwan Co. Ltd. and randomly divided into three groups consisting of seven animals each. All animals were kept in a room maintained at 24°C ± 1°C and humidity of 55% ± 10% with a 12‐h light/dark cycle. During the study, these rats were fed rodent chow (Lab Diet, 5001, PMI Feeds Inc., Richmond, IN, USA) and drank sterile water ad libitum. 5FU was purchased from Nang Kuang Pharmaceutical Co. Ltd. The original concentration of 5FU was 50 mg/mL and was diluted to 10 mg/mL with phosphate‐buffered saline (PBS).

### 2.2 Ethics Statement

All animal experimental procedures were reviewed and approved by the Animal Care and Use Committee of Chung Shan Medical University, Taiwan, in accordance with the 3R principles (Replacement, Reduction, and Refinement) (Approval No: 2282).

We appreciate your guidance on this important aspect.

### 2.3 Probiotic Preparation

The probiotic strains *L. reuteri* and *C. butyricum* Miyairi 588 (CBM588), investigated in this study, were selected based on their demonstrated increased gut microbiota abundance in a cisplatin‐induced nephrotoxicity model when coadministered with D‐methionine (Wu et al. 2019). The nephroprotective effects of LCs are associated with their ability to enhance anti‐inflammatory responses and maintain the integrity of the intestinal barrier (Hsiao et al. 2021). Additionally, both strains have been reported to exhibit antioxidant and anti‐inflammatory effects in the intestinal environment (Ariyoshi et al. 2021; Yue et al. 2025). Based on these findings, both bacterial strains, as potential probiotics, were selected for further investigation to determine whether the administration of LCs could alleviate diarrhea induced by 5FU through the regulation of intestinal barrier integrity.

The probiotic mixtures used in this study were *L. reuteri and C. butyricum* (Miyarisan Pharmaceutical). *L. reuteri* (BCRC 80379) was obtained from the Food Industry Research and Development Institute (Hsinchu, Taiwan), while CBM588 was purchased from Asia Bio‐Med Management Limited (Taichung, Taiwan). The total quantity of bacteria CBM588 powder was 5 × 10^8^ cfu/g/packet. In an anaerobic incubator, *L. reuteri* cultures were grown overnight in Lactobacilli MRS broth at 37°C. the culture broth was centrifuged at 2000 × g for 3 min and washed three times with PBS. The probiotic mixtures contained viable CBM588 at 5 × 10^8^ colony‐forming units (CFU)/g and viable *L. reuteri* at 1–2 × 10^9^ CFU/mL and were reconstituted in 2 mL sterile saline before administration. Control mice were treated with the same volume of sterile water.

### 2.4 Study Design and Sample Treatment

After a week of preexperimental adaptation, the rats of three groups were given different treatments: control group, 5FU group, 5FU + *L. reuteri* , and *C. butyricum* (5FU + LCs group). To establish an intestinal mucositis model, we adapted a protocol from a previous study (Chen et al. 2021) with minor modifications. 5FU was administered via intraperitoneal injection at a dose of 50 mg/kg body weight from day 1 to day 5. In the control group, rats received physiological saline injections during the same period, along with daily oral administration of sterile water by gavage. The 5FU group was similarly treated with 5FU and daily oral sterile water by gavage from day 1 to day 5. The 5FU + LCs group received probiotic supplementation for 14 days before the initial 5FU injection and continued with five intraperitoneal injections of 5FU from day 1 to day 5. Throughout the study, the body weight, food intake, water consumption, and stool characteristics of the rats were monitored daily. The diarrhea of all animals was observed every day and was graded accordingly as follows: 0, normal stool (normal); 1, considerably moist stool (mild); 2, unformed and wet stool (moderate); and 3, watery stool (severe) (Kim et al. 2018).

All rats were anesthetized with CO_2_ and were sacrificed on the 8th day. The colons (from the ileocecal junction to the anorectal junction) were removed, and colon length was measured with a ruler. Then the colons were immediately divided into two portions: one kept in 10% formalin for histological and immunohistochemical evaluation. Another half of the tissues was frozen in liquid nitrogen and stored at −80°C until used for the preparation of tissue homogenates, real‐time PCR, and Western blot analysis.

### 2.5 Histopathological Examination of Colon

The colon tissues were fixed in 10% neutral buffered formalin, dehydrated in graded ethanol, embedded in paraffin wax, processed, sectioned into 4 mm‐thick slices, and stained with hematoxylin and eosin (H&E). The histological injury score of each colon was evaluated by experienced pathologists with light microscopy (Olympus, Japan) at a magnification of 40X, 100X, and 400X.

### 2.6 Preparation of Tissues Homogenates

The colons (approximately 0.1 g) were homogenized in 10 mL of cold PBS buffer using a homogenizer at 4°C. The crude tissue homogenate was centrifuged and kept at −80°C for further use.

### 2.7 Oxidative Stress Biomarkers Analysis

Malondialdehyde (MDA) levels in colon homogenates were determined using the thiobarbituric acid (TBA) reaction method (Wen et al. 2004). The standard substance used was 1,1,3,3‐tetramethoxypropane (TMP). TMP was used as the standard for calibration, with concentrations of 0, 0.2, 1, 2, 5, and 10 nmol/mL. Colon homogenates were diluted 1:1 with phosphate‐buffered saline (PBS). For the assay, 500 μL of each sample or standard was mixed with 500 μL of 40% trichloroacetic acid (TCA) to precipitate proteins. Subsequently, 0.85% TBA was added, and the mixture was thoroughly vortexed and incubated in a boiling water bath (100°C) for 20 min. Following cooling, 1 mL of the reaction mixture was transferred to a 1.5 mL microcentrifuge tube and centrifuged. The supernatant was collected, and its absorbance was measured at 532 nm. The MDA concentration was calculated based on the standard curve.

Glutathione peroxidase (GPx) activity was determined by monitoring the oxidation of reduced glutathione (GSH) coupled with the reduction of nicotinamide adenine dinucleotide phosphate (NADPH) by glutathione reductase (GSR), as previously described (Wen et al. 2004). In brief, 10 μL of colon homogenate was mixed with 50 mM phosphate buffer (PPB) and 30 mM hydrogen peroxide (H_2_O_2_). The reaction was monitored using a UV–visible spectrophotometer (Ultrospec 2100 pro, Biosciences Amersham).

The decomposition rate of H_2_O_2_ at 240 nm was measured to assess catalase activity (Wen et al. 2004). Briefly, 10 μL of diluted homogenate was mixed with 690 μL of 50 mM PPB and 350 μL of 30 mM H_2_O_2_. After rapid mixing, the decline in absorbance at 240 nm was recorded within 1 min.

The concentrations of interleukin‐10 (IL‐10) and interleukin‐1β (IL‐1β) in colon homogenates were quantified using rat ELISA kits (R&D Systems, Minneapolis, MN, USA), according to the manufacturer's protocols.

### 2.8 Measurement of Diamine Oxidase (DAO) and Myeloid Peroxidase Enzyme (MPO)

DAO activity in the colons homogenates was determined using the commercial rat diamino oxidase ELISA kit (CSB‐E12634r, Cusabio Biotech, Wuhan) according to the protocols of the ELISA kits. The MPO activity in the colon tissue samples was determined according to the previous method with some alteration (Kuo et al. 2011). The supernatant (50 μL) was added to 1.4 mL of 50 mM phosphate buffer containing O‐dianisidine dihydrochloride and 50 μL H_2_O_2_. Finally, the absorbance changes were recorded at a wavelength of 460 nm within 3 min using the Amersham‐Ultrospec 2100 Pro spectrophotometer (LSI Model Alfa‐1502).

### 2.9 RNA Extraction and Real‐Time Reverse Transcriptase‐PCR (Real‐Time RT‐PCR)

Total RNA was extracted from colon tissues using RareRNA (GenePure Technology Co., Taichung, Taiwan) according to the manufacturer's protocol. The concentration of each RNA sample and the 260/280‐nm absorbance (A260/A280) ratio were measured using the Amersham‐Ultrospec 2100 Pro Spectrophotometer. Purified RNA (3 μg/μL) was reverse‐transcribed into cDNA using a High Capacity cDNA Reverse Transcription Kit (Applied Biosystems, Foster City, CA). RT‐qPCR was carried out on a StepOne Real‐Time PCR System (Applied Biosystems, Foster City, USA) using the Smart Quant Green Master Mix (Applied Biosystems). PCR was performed with 1 ⎧λ sample cDNA, Smart Quant Green Master Mix 5 μL, 1 μLof each primer of GAPDH, and 1 μL of each primer of the gene to be tested, in a final volume of 10 μL. Ct values for both the target and internal control genes were calculated, and the relative changes in gene expression were analyzed by the 2^−ΔΔCt^ method. The mRNA of GAPDH was used to normalize the total amount of cDNA on real‐time PCR. The primer sequences for PCR amplification are presented in Table 1.

**TABLE 1:** Primer sequences for RT‐qPCR.

Target	Sequence (5′ → 3′)	Accession
TNF‐α	Forward CCCAATCTGTGTCCTTCTAACT	[ΝΜ_012675.3]
	Reverse CACTACTTCAGCGTCTCGTGT	
IFN‐γ	Forward CACGCCGCGTCTTGGT	[ΝΜ_138880.3]
	Reverse TCTAGGCTTTCAATGAGTGTGCC	
iNOS	ForwardACAACGTGGAGAAAACCCCAGGTG	[ΝΜ_012611.3]
	Reverse ACAGCTCCGGGCATCGAAGACC	
VCAM‐1	Forward AAGTGGAGGTCTACTCATTCC	[ΝΜ_012889.2]
	Reverse GGTCAAAGGGGTACACATTAG	
Occludin	Forward TTGGGAGCCTTGACATCTTGTTC	[XM_039103245.1]
	Reverse GCCATACATGTCATTGCTTGGTG	
CXCL‐4	Forward TTCTTCTGGGTCTGCTGTTG	[ΝΜ_001007729.1]
	Reverse TGCGTTTGAGATGGATCCTG	
Caspase‐3	Forward GAGACAGACAGTGGAACTGACGATG	[XΜ_006253130.4]
	Reverse GGCGCAAAGTGACTGGATGA	
AQP‐7	Forward GCTGGCTGGGGCAAGAAAGTG	[XΜ_039109352.1]
	Reverse TTTATTGCAGAAGGGTTGTGGTCA	
VIP	Forward TCTGCAAGGGTAGCAATCGA	[XΜ_039078200.1]
	Reverse GGTGGAGTCCCTATCACTGG	
MAPK	Forward GGGTCGTGGTACTGAGCAAA	[ΝΜ_031020.3]
	Reverse ATAATGCGTCTGACGGGGAC	
NFκB	Forward GCAACTCTGTCCTGCACCTA	[ΝΜ_001276711.1]
	Reverse CTGCTCCTGAGCGTTGACTT	

### 2.10 The Analysis of the Short‐Chain Fatty Acids (SCFAs) in Cecum Content

The fecal short‐chain fatty acid extraction and analysis method is as follows. Approximately 0.5 g of rat fecal samples was weighed and transferred to 15 mL centrifuge tubes. Deionized water (5 mL) was added, and the samples were homogenized for 2 min. The homogenates were then centrifuged at 7000 rpm for 5 min at 25°C, and the supernatants were filtered. An aliquot of 2 mL of the supernatant was collected and spiked with a standard isocaproic acid solution. Subsequently, 200 μL of 50% (v/v) sulfuric acid was added, followed by 2 mL of diethyl ether. The mixture was homogenized for 2 min and then centrifuged at 4000 rpm for 5 min at 25°C. Collect the supernatant for subsequent gas chromatography with flame ionization detection (GC/FID) analysis to quantify the SCFA profiles.

### 2.11 Statistical Analyses

A one‐way analysis of variance (ANOVA) was conducted using IBM SPSS Statistics 20, followed by Tukey's honestly significant difference test (Tukey's HSD) for post hoc analysis. Descriptive statistics were expressed as means ± standard error of the mean (SEM). All statistical results were considered significant at *p* < 0.05.

## 3 Results

### 3.1 Changes in Diarrhea, Disease Activity Index (DAI), and Colon Length in Rats With 5FU‐Induced Colitis

**FIGURE 1:**
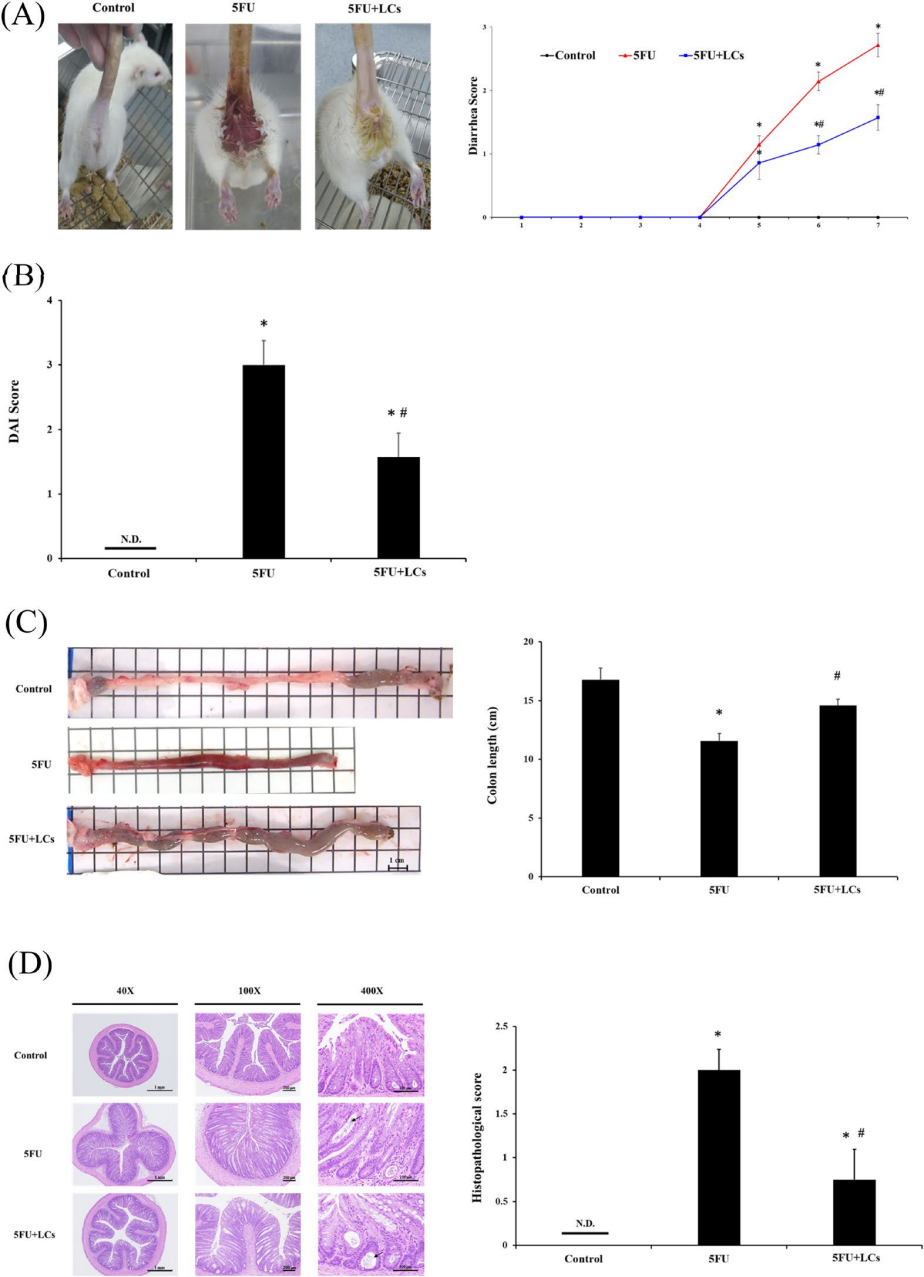
LCs alleviate 5‐FU‐induced colitis. Assessment of diarrhea, colon length, and colitis severity across the three experimental groups. (A) Photographs of diarrhea in rats and their corresponding diarrhea scores, (B) Disease activity index (DAI) score, (C) Representative image of the colon and lengths of the colons, (D) HE staining of colonic sections under original magnification: 40X, 100X and 400X (black arrow showed multifocal cryptic apoptosis and dilation) and (E) The histopathological score of colon tissue. Data are presented as mean ± SEM, *n* = 7. Differences were analyzed by one‐way ANOVA. The data were statistically analyzed, with * denoting a significant difference compared to the control group (*p* < 0.05), and # indicating a significant difference compared to the 5FU‐treated group (*p* < 0.05).

Diarrhea is a well‐documented adverse effect of 5FU treatment. To evaluate the potential protective effect of probiotic LCs against 5FU‐induced colitis, the incidence of diarrhea was first assessed. As illustrated in Figure 1A, no significant diarrhea was observed in the control group throughout the experimental period. In contrast, rats treated with 5FU exhibited a marked increase in diarrhea incidence starting from day 4, with some animals showing bloody stools. Notably, coadministration of LCs significantly reduced the occurrence of diarrhea compared to the 5FU‐only group (*p* < 0.05). Colitis severity was further evaluated using the DAI, which integrates scores for stool consistency, fecal occult blood, and body weight loss (Sang et al. 2013). Treatment with 5FU significantly elevated DAI values compared to the control group (*p* < 0.05). However, probiotic supplementation with LCs markedly attenuated the DAI scores in comparison to the 5FU group (Figure 1B). Similarly, the diarrhea scores were significantly lower in the 5FU + LCs group than in the 5FU group. In addition, since severe diarrhea is often associated with colonic shortening (Chen et al. 2022), colon length was measured at the end of the experiment. Rats exposed to 5FU exhibited a significant reduction in colon length after 5 days of consecutive administration, while cotreatment with LCs effectively mitigated this shortening effect (Figure 1C).

### 3.2 Histopathological Changes in the Colon Following 5FU Treatment

Histological evaluation of colonic tissues was performed to assess mucosal damage induced by 5FU. The scoring criteria included crypt cell apoptosis and expansion, mucosal atrophy and inflammation, as well as the reduction of goblet cells. As shown in Figure 1D,E, the 5FU‐treated group exhibited a significant increase in histopathological scores compared to the control group (*p* < 0.05), indicating substantial tissue damage. Notably, LCs supplementation markedly alleviated the severity of these histological lesions; however, the protective effect did not fully restore the colonic architecture to the level observed in the control group.

### 3.3 Effects of 5FU and LCs on Oxidative Stress Markers in Colon Tissue

**FIGURE 2:**
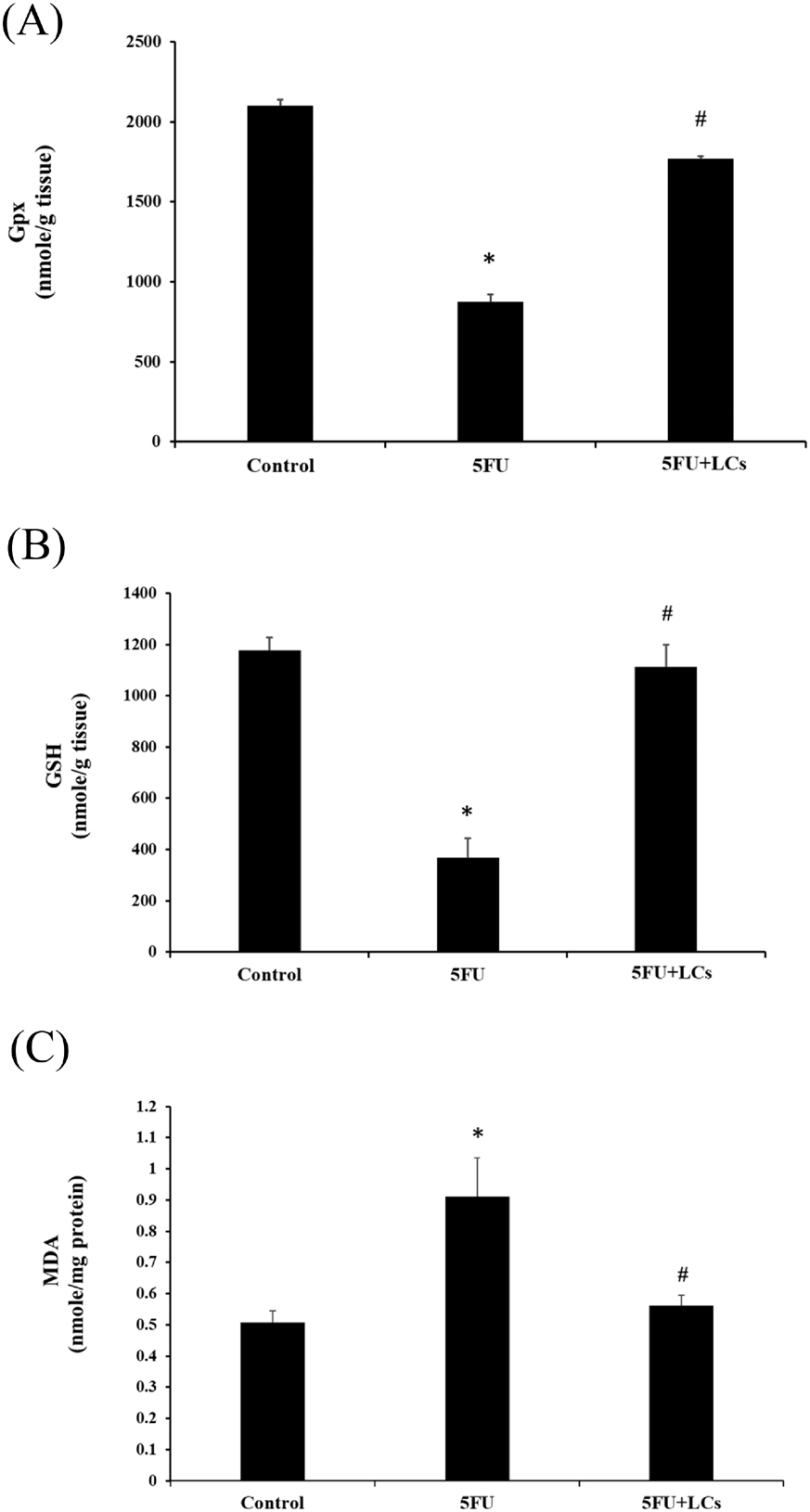
Effect of LCs on oxidative stress in 5FU‐induced colitis. (A) GPx activity, (B) GSH level, and (C) Lipid peroxidation (MDA level). Data are presented as mean ± SEM, *n* = 7. Differences were analyzed by one‐way ANOVA. The data were statistically analyzed, with * denoting a significant difference compared to the control group (*p* < 0.05), and # indicating a significant difference compared to the 5FU‐treated group (*p* < 0.05).

To investigate oxidative stress status, the concentrations of MDA as an index of lipid peroxidation and GSH, along with GPx activity as indicators of antioxidant capacity, were measured in colonic tissues (Figure 2A–C). Treatment with 5FU significantly increased MDA levels while reducing both GSH concentrations and GPx activity compared to the control group (*p* < 0.05), indicating enhanced oxidative damage. In contrast, pretreatment with LCs effectively mitigated these alterations, restoring MDA, GSH, and GPx levels toward values comparable to those of the control group.

### 3.4 Effect of LCs on Inflammatory Markers in 5FU‐Induced Colon Tissue

**FIGURE 3:**
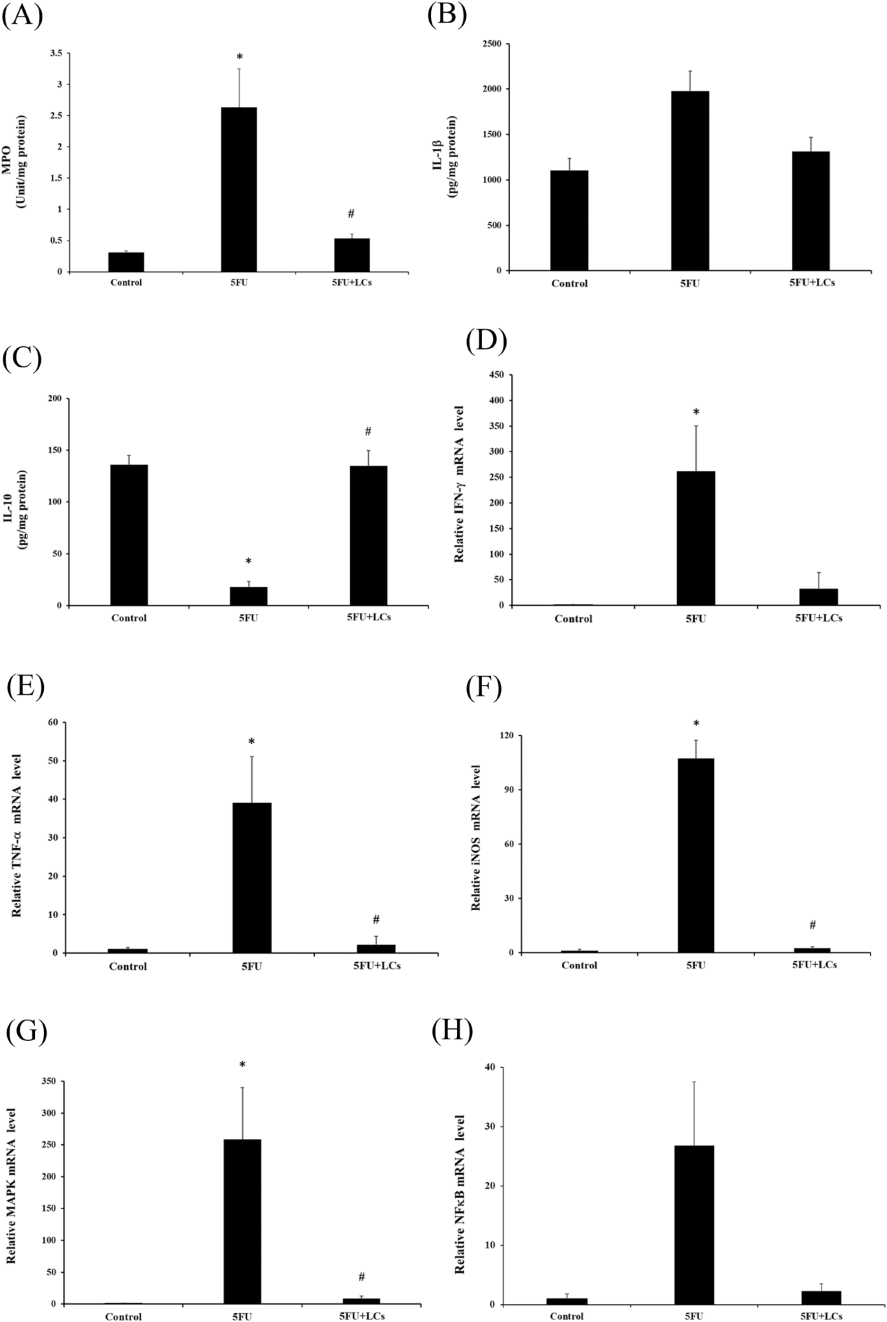
The effect of LCs on 5‐FU‐induced inflammatory cytokines in colonic tissues at both protein production and gene expression levels. (A) Myeloperoxidase (MPO) activity and cytokine concentrations, (B) IL‐1β levels; (C) IL‐10 levels. The gene expression levels of (D) IFN‐γ, (E) TNF‐α, (F) iNOS, and (G) NF‐κB. Data are presented as mean ± SEM, *n* = 3–7. Differences were analyzed by one‐way ANOVA. The data were statistically analyzed, with * denoting a significant difference compared to the control group (*p* < 0.05), and # indicating a significant difference compared to the 5FU‐treated group (*p* < 0.05).

Previous studies have demonstrated that increased leukocyte infiltration correlates with the severity of colitis, as observed in DSS‐induced animal models (Sang et al. 2013). In the present study, a significant elevation in myeloperoxidase (MPO) activity, a marker of neutrophil infiltration, was detected in the colonic tissue of rats following five consecutive days of 5FU administration compared to the control group (*p* < 0.05, Figure 3A). Notably, coadministration of LCs significantly attenuated this increase, indicating a reduction in leukocyte infiltration into the colonic mucosa. Inflammatory cytokines are recognized as pivotal mediators in the progression of colitis‐associated tissue damage. As shown in Figure 3B, IL‐1β levels were significantly elevated in the colonic tissues of the 5FU group relative to controls (*p* < 0.05). Although IL‐1β concentrations in the LCs‐treated group showed a decreasing trend compared to the 5FU group, this difference did not reach statistical significance (*p* > 0.05). Conversely, IL‐10, an anti‐inflammatory cytokine, was significantly suppressed in the 5FU group compared to controls, while LCs supplementation effectively restored IL‐10 levels (*p* < 0.05, Figure 3C). Additionally, the mRNA expression of several pro‐inflammatory markers, including IFN‐γ, TNF‐α, iNOS, MAPK, and NF‐κB, was analyzed in colonic tissues. As illustrated in Figure 3D–G, 5FU treatment significantly upregulated IFN‐γ, TNF‐α, iNOS, and MAPK expression compared to the control group (*p* < 0.05). Supplementation with LCs markedly suppressed TNF‐α, iNOS, and MAPK expression levels (*p* < 0.05). Although IFN‐γ expression was reduced in the LCs group relative to the 5FU group, the difference was not statistically significant. NF‐κB expression (Figure 3H) was elevated following 5FU treatment but did not differ significantly among the experimental groups. Collectively, these results suggest that LCs supplementation partially alleviates 5FU‐induced colonic inflammation by modulating the expression of inflammatory mediators and limiting leukocyte infiltration.

### 3.5 LCs Protect the Integrity of the Intestinal Barrier

**FIGURE 4:**
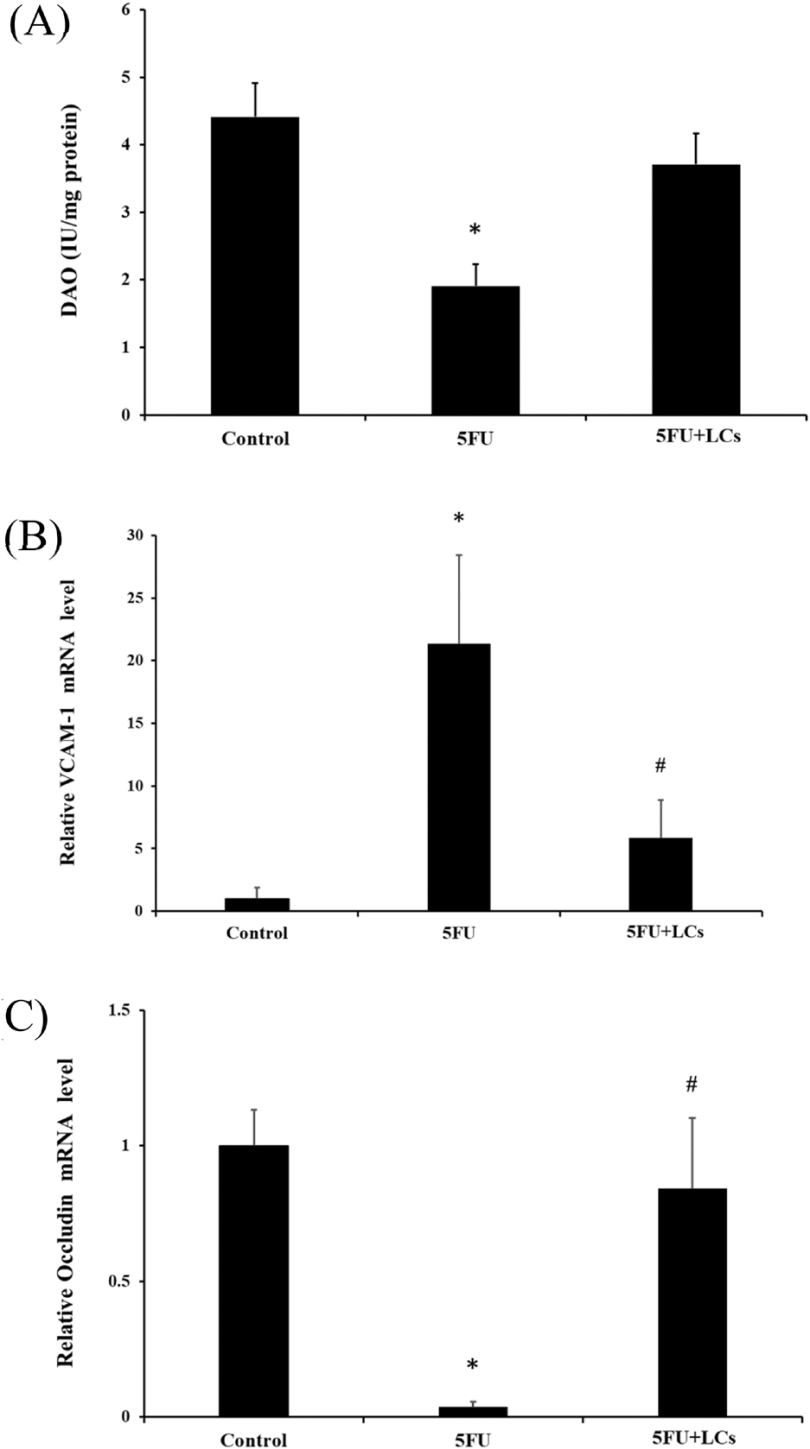
Effect of LCs on intestinal mucosal integrity in rats treated with 5FU. (A) Diamine oxidase (DAO) activities; (B) gene expression levels of the inflammatory marker VCAM‐1; (C) gene expression levels of occludin, as measured by quantitative PCR (qPCR) assay. Data are presented as mean ± SEM, *n* = 3–7. Differences were analyzed by one‐way ANOVA. The data were statistically analyzed, with * denoting a significant difference compared to the control group (*p* < 0.05), and # indicating a significant difference compared to the 5FU‐treated group (*p* < 0.05).

DAO activity is considered a reliable biomarker of intestinal permeability, with decreased DAO activity reflecting increased barrier dysfunction (Zheng et al. 2023). As shown in Figure 4A, 5FU treatment significantly reduced DAO activity compared to the control group (*p* < 0.05). However, LCs supplementation markedly restored DAO activity in 5FU‐treated rats. In addition, the gene expression of VCAM‐1 and occludin, a key tight junction protein, was assessed by real‐time PCR. As illustrated in Figure 4B,C, 5FU administration significantly elevated VCAM‐1 expression while suppressing occludin expression, indicating disruption of the intestinal barrier. Notably, LCs supplementation significantly downregulated VCAM‐1 and upregulated occludin expression in 5FU‐treated rats (*p* < 0.05), suggesting that LCs contribute to the preservation of intestinal barrier integrity under chemotherapeutic stress.

### 3.6 Effect of LCs on the Apoptosis in 5FU‐Treated Colon Tissue

**FIGURE 5:**
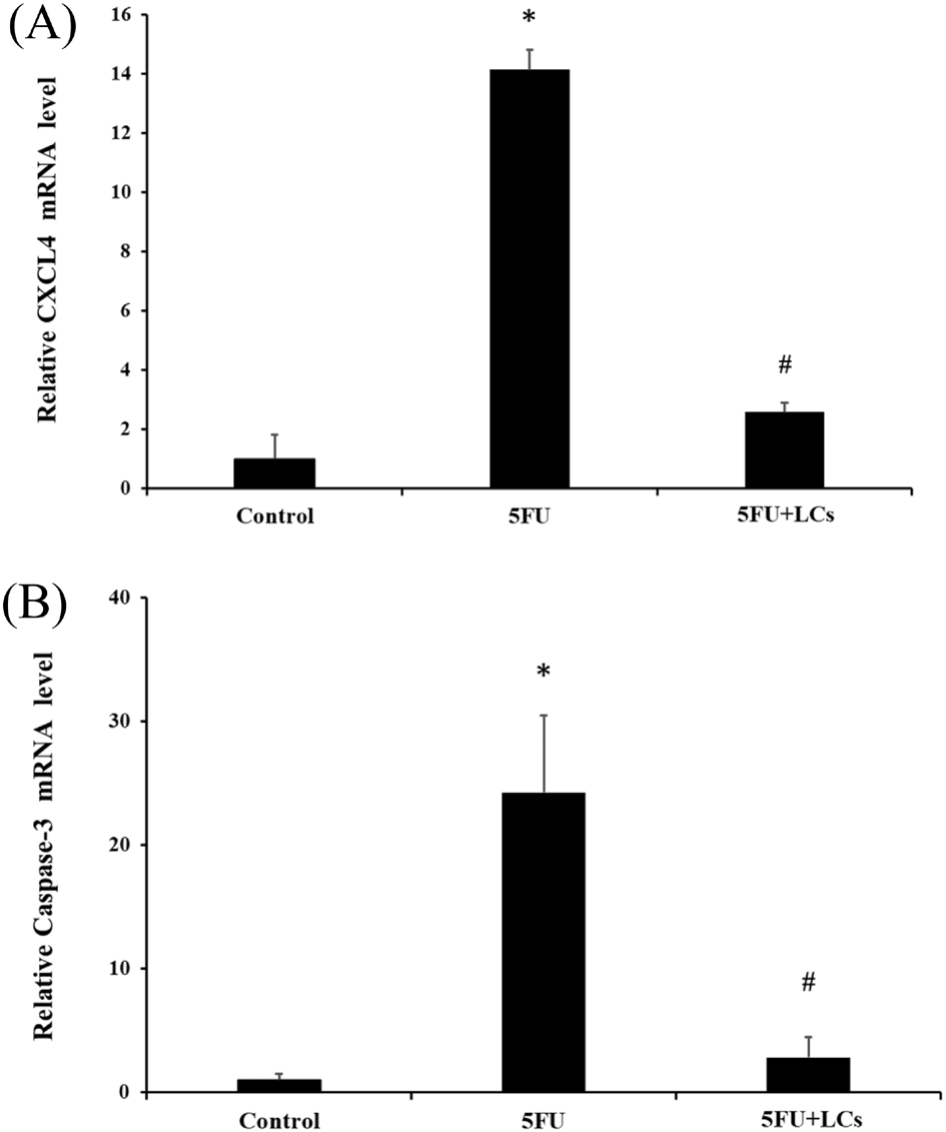
Effect of LCs on apoptosis‐related inducer in 5FU induced colonic tissues. The gene expression levels of (A) CXCL 4 and (B) caspase‐3, as measured by qPCR assay. Data are presented as mean ± SEM, *n* = 3. Differences were analyzed by one‐way ANOVA. The data were statistically analyzed, with * denoting a significant difference compared to the control group (*p* < 0.05), and # indicating a significant difference compared to the 5FU‐treated group (*p* < 0.05).

Apoptosis is a key mechanism of 5FU‐induced mucosal injury, partly mediated by the upregulation of chemokine CXCL4 (Gao et al. 2014). Given the protective effect of LCs on colitis severity, we further evaluated their influence on apoptosis‐related gene expression. As shown in Figure 5, the mRNA levels of CXCL4 and caspase‐3 were significantly elevated in the colonic tissues of 5FU‐treated rats compared to controls (*p* < 0.05). Importantly, LCs supplementation significantly attenuated the expression of both CXCL4 and caspase‐3, indicating that LCs may mitigate 5FU‐induced mucosal apoptosis (Figure 5).

**FIGURE 6:**
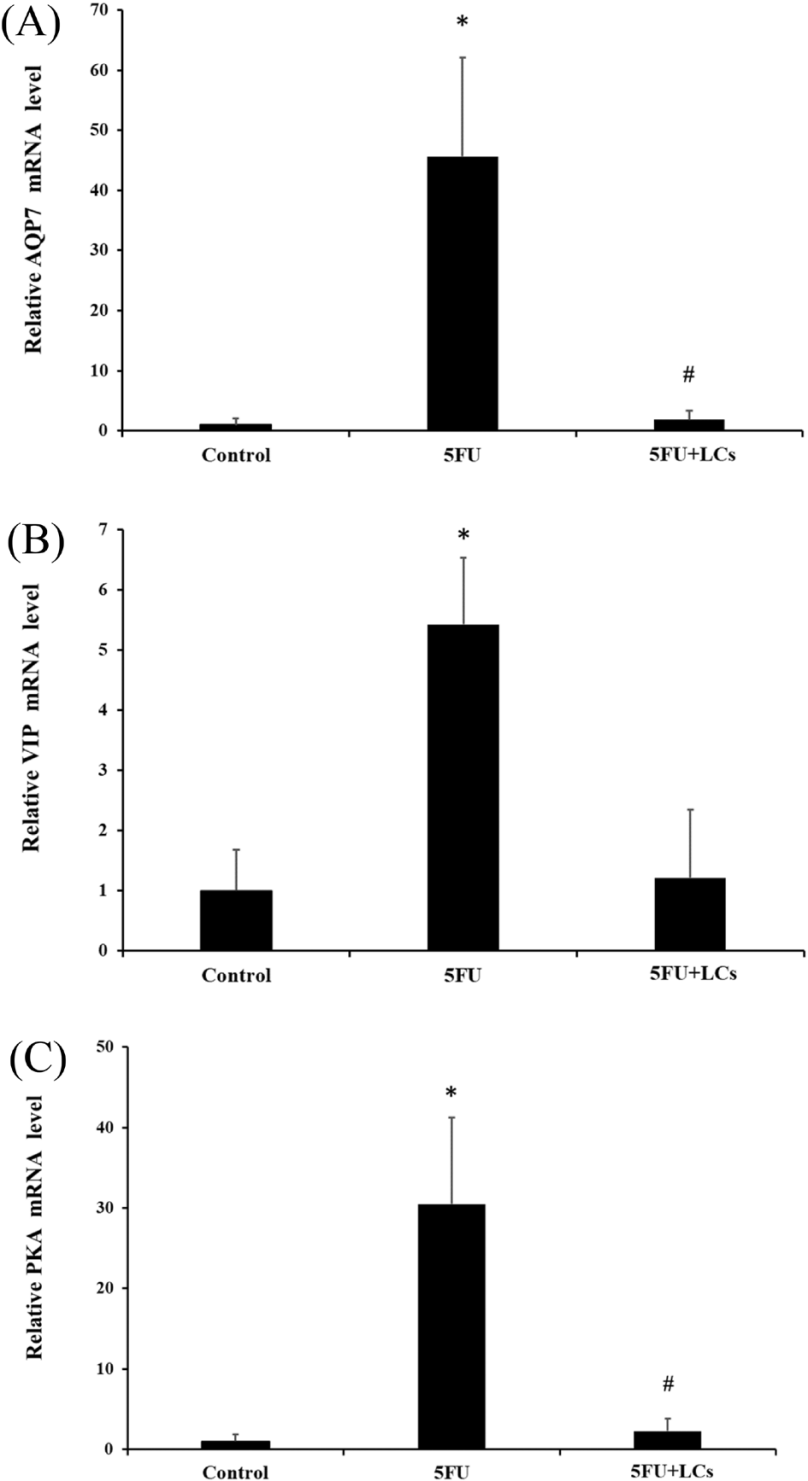
Effect of LCs on water transport in rats treated with 5‐FU. The mRNA expression levels were analyzed to assess the impact of LCs treatment on key transport proteins. (A) AQP7 is a water channel protein that plays a crucial role in water reabsorption in the intestines; (B) VIP is involved in regulating water and electrolyte secretion, which may affect fluid balance; (C) PKA is a critical enzyme involved in various signaling pathways that can influence cellular transport processes. Data are presented as mean ± SEM, *n* = 3. Differences were analyzed by one‐way ANOVA. The data were statistically analyzed, with * denoting a significant difference compared to the control group (*p* < 0.05), and # indicating a significant difference compared to the 5FU‐treated group (*p* < 0.05).

### 3.7 Effect of LCs on the Aquaporins (AQPs) in 5FU‐Induced Colitis

Given the observed alleviation of diarrhea and barrier dysfunction by LCs, we further investigated their impact on the gene expression of aquaporin 7 (AQP7), vasoactive intestinal peptide (VIP), and protein kinase A (PKA). As shown in Figure 6, 5FU treatment significantly upregulated AQP7, VIP, and PKA mRNA levels compared to controls (*p* < 0.05). Interestingly, LCs supplementation significantly suppressed the expression of AQP7 and PKA (*p* < 0.05) but had no significant effect on VIP expression. These findings suggest that the anti‐diarrheal effects of LCs may be partly mediated through the regulation of water transport and signaling pathways in the colon.

**FIGURE 7:**
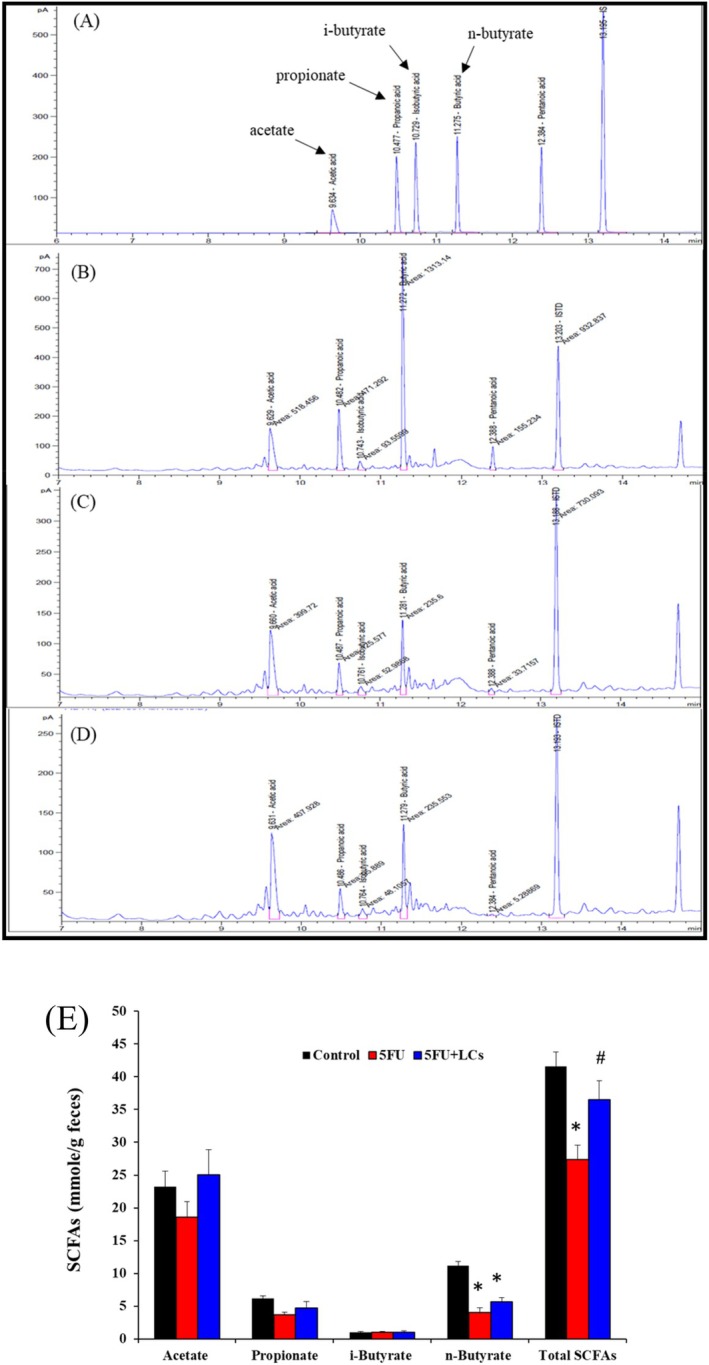
Concentrations of short‐chain fatty acids (SCFAs) in the fecal samples of rats from different experimental groups at the end of the experiment. SCFAs, including acetate, propionate, and butyrate, are crucial metabolites produced by the fermentation of dietary fibers by gut microbiota. The Gas chromatography profile of (A) Standard spectrum of short‐chain fatty acids, (B) Control group, (C) 5FU group, (D) 5FU+ LCs group, and (E) concentration of SCFAs among the three experimental groups. Total SCFAs = Acetic + Propionic + isobutyric acid + Butyric. Data are presented as mean ± SEM, *n* = 7. Differences were analyzed by one‐way ANOVA. The data were statistically analyzed, with * denoting a significant difference compared to the control group (*p* < 0.05), and # indicating a significant difference compared to the 5FU‐treated group (*p* < 0.05).

### 3.8 The Concentrations of SCFAs

SCFAs are known to play an essential role in maintaining intestinal health by reducing mucosal inflammation (Shen et al. 2021). To evaluate whether LCs supplementation could modulate SCFA production under 5FU‐induced colitis, the concentrations of specific short‐chain fatty acids, including acetic acid, propionic acid, isobutyric acid, and butyric acid, were quantified in fecal samples using GC‐FID. The retention times (RT) for each compound were: acetic acid (9.6 min), propionic acid (10.5 min), isobutyric acid (10.7 min), and butyric acid (11.3 min). SCFA levels were calculated based on peak area comparisons with standard curves (Figure 7A). As shown in Figure 7E, the concentrations of acetic acid, propionic acid, and isobutyric acid did not differ significantly among the control, 5FU, and 5FU + LCs groups. However, butyric acid levels were significantly reduced in both the 5FU and 5FU + LCs groups compared to the control group (*p* < 0.05). Although LCs supplementation slightly increased butyric acid levels compared to the 5FU group, the difference did not reach statistical significance (*p* > 0.05). Furthermore, the total SCFA concentration was markedly decreased in the 5FU group relative to controls (*p* < 0.05). Notably, LCs supplementation significantly restored total SCFA levels in the 5FU + LCs group compared to the 5FU group (*p* < 0.05), suggesting that LCs may contribute to gut homeostasis through modulation of SCFA production.

## 4 Discussion

5‐U is a chemotherapy agent utilized in cancer treatment; however, it negatively impacts rapidly dividing cells, including those in the gastrointestinal epithelium. This can result in a range of gastrointestinal issues, notably intestinal mucositis. Clinically, the diarrhea caused by 5FU is especially worrisome (Yeung et al. 2021). Diarrhea results from damage to the intestinal mucosa, which initiates repeated apoptosis and inflammation of the intestinal epithelium and intestinal wall. Chemotherapy‐induced diarrhea can be life‐threatening due to ongoing electrolyte and fluid losses, compounded by malnutrition. In this study, rats receiving 5FU for 5 days exhibited moderate to severe diarrhea. However, LCs treatment mitigated the incidence of diarrhea (Figure 1A). Furthermore, intestinal mucosal injury, bleeding, and inflammation can lead to shortening of the colon. The DAI and colon length are important indicators for evaluating the severity of colitis in mice (Yue et al. 2025). Consistent with our findings (Figure 1B,C), LCs demonstrated the ability to reduce DAI scores and attenuate colon shortening, indicating their therapeutic potential against colitis‐related pathological effects. Probiotics have a longstanding history in the treatment of diverse intestinal diseases, encompassing conditions such as diarrhea and enteritis. CBM588 has shown effectiveness in not only preventing ulcerative colitis and exhibiting antitumor properties but also in significantly reducing diarrhea and safeguarding the intestinal barrier (Ariyoshi et al. 2021; Hagihara et al. 2020). In a study on diarrhea induced by *Escherichia coli* in pigs, the addition of *L. reuteri* LR1 notably decreased both diarrhea incidence and intestinal permeability (Tang et al. 2021). *L. reuteri* significantly alleviated DSS‐induced reduced shortening of colon length and effectively prevented the increase of DAI (Yue et al. 2025). These results suggest that probiotics have a protective effect against colitis.

It is well established that lactic acid bacteria (LAB) exert antioxidant effects through the production of bioactive metabolites, surface‐associated molecules, and specific antioxidant enzymes. Probiotics are capable of synthesizing or secreting antioxidant components, including extracellular polysaccharides (EPS), superoxide dismutase (SOD), GPx, catalase (CAT), pseudocatalase, SCFAs, and tryptophan derivatives, all of which contribute to alleviating oxidative stress and inflammatory responses in host tissues (Feng and Wang 2020; García Mansilla et al. 2025; Wu, Xie, et al. 2022). Specifically, *L. reuteri* has been shown to produce antioxidant enzymes such as SOD and CAT (Jalali et al. 2024). Moreover, genome sequencing analyses have confirmed the presence of genes encoding oxidases, reductases, and dehydrogenases, suggesting its potential regulatory role in oxidative stress (Ali et al. 2023). In addition to enzymes, *L. reuteri* is recognized as a significant producer of EPS, which has been reported to mitigate oxidative stress and inflammation by modulating immune responses and enhancing antioxidant enzyme activities including SOD, GPx, and CAT (Ferrer et al. 2024; Ksonzekova et al. 2016; Liu, Cheng, et al. 2024). Another probiotic used in this study, *C. butyricum* , is a known butyrate‐producing bacterium, and its administration has been demonstrated to activate the Keap1‐Nrf2/HO‐1 pathway, leading to the transcription of antioxidant genes such as HO‐1 and reinforcing the cellular antioxidant defense (Zhou et al. 2024). Additionally, SCFAs derived from *C. butyricum* and *L. reuteri* can further activate Nrf2 signaling, thereby boosting antioxidant enzyme expression and minimizing oxidative injury (Liu, Cheng, et al. 2024). Recent studies have also shown that probiotic combinations such as *L. plantarum* AS21 and *C. butyricum* can enhance microbial taxa linked to antioxidant and anti‐inflammatory benefits (Li et al. 2024). Furthermore, clinical investigations have reported that probiotic supplementation during chemotherapy not only alleviates mucositis but also restores SCFA‐producing microbiota, further contributing to intestinal antioxidant defense (Huang et al. 2023).

SCFAs have also been shown to stimulate the synthesis and activity of antioxidant enzymes, providing additional protection against oxidative stress (Liu, Cheng, et al. 2024). Taken together, the antioxidant properties of *L. reuteri* and *C. butyricum* appear to involve multiple complementary mechanisms, including the production of antioxidant enzymes (SOD, GPx, CAT), EPS, and SCFAs, as well as the activation of the Keap1–Nrf2 signaling pathway. These combined actions help alleviate oxidative stress, preserve redox balance, and protect the integrity of the intestinal mucosa during chemotherapy‐induced injury. While the antioxidant activity of probiotics may involve multiple mechanisms, these processes are not yet fully understood and warrant further investigation.

The intestinal barrier is a dynamic epithelial structure that facilitates nutrient absorption and waste excretion while preventing the translocation of harmful luminal substances. Tight junction (TJ) proteins, such as claudin‐1, occludin, and ZO‐1, are essential for maintaining epithelial integrity. Inflammatory cytokines and oxidative stress can impair TJ protein expression, resulting in increased intestinal permeability and barrier dysfunction (Yong et al. 2021; Yu et al. 2024). Cell adhesion proteins, such as selectins, integrins, ICAM‐1, and VCAM‐1, play crucial roles in mediating the adhesion and migration of leukocytes, as well as in regulating immune responses and inflammatory processes. Among these, ICAM‐1 is particularly significant in orchestrating the recruitment of white blood cells to sites of tissue injury, whereas the expression of VCAM‐1 is markedly upregulated in response to inflammatory stimuli. Consequently, both ICAM‐1 and VCAM‐1 serve as important biomarkers for evaluating intestinal mucosal lesions (Wu, Jha, et al. 2022). Chemotherapy agents like 5FU have been shown to disrupt intestinal barrier integrity by downregulating TJ proteins (occludin, claudin‐1) and upregulating adhesion molecules (VCAM‐1, ICAM‐1, JAM‐A), ultimately contributing to diarrhea (Li et al. 2017; Zheng et al. 2023). Elevated TNF‐α levels can induce intestinal epithelial apoptosis through caspase‐3 activation, further compromising barrier integrity (Geng et al. 2024). Probiotics such as *L. plantarum* HNU082 have been reported to restore the expression of TJ proteins and mucin‐2 and suppress the expression of adhesion molecules, thereby improving barrier function and reducing inflammation in colitis models (Wu, Jha, et al. 2022). Interestingly, a recent meta‐analysis also reported that probiotic supplementation, particularly multispecies formulations, longer intervention durations (≥ 12 weeks), and applications in patients with metabolic syndrome resulted in a significant reduction of circulating VCAM‐1 levels, suggesting a systemic anti‐inflammatory effect beyond the gut (Rezazadeh et al. 2023). This finding echoes our current hypothesis that combined probiotic supplementation, such as LCs, may mitigate neutrophil adhesion by downregulating adhesion molecules like VCAM‐1, prevent TJ protein loss, suppress epithelial apoptosis, and enhance DAO activity. Collectively, these interlinked effects contribute to reducing colonic epithelial permeability and maintaining barrier integrity, further highlighting the potential of probiotics in modulating intestinal barrier function under inflammatory conditions.

Diamine oxidase (DAO) is an enzyme predominantly localized in the mucosal villous epithelial cells, and its activity markedly decreases during intestinal mucositis, reflecting compromised mucosal integrity. Furthermore, necrosis of intestinal mucosal cells can result in their detachment and subsequent shedding into the intestinal lumen, which contributes to reduced levels of DAO within the intestinal mucosa and an elevation of circulating DAO levels (Shi et al. 2022). Consequently, mucosal DAO activity is regarded as an indicator of intestinal mucosal maturation and integrity, as well as a marker for mucosal injury and recovery. Additionally, DAO activity was found to be diminished in mice exhibiting mucosal impairment induced by 5FU treatment. The observed increase in intestinal DAO levels suggests a restoration of tight junction integrity following the damage incurred from chemotherapy‐induced intestinal mucosal injury (Zheng et al. 2023). Our study further demonstrated that 5FU‐induced intestinal injury led to an increase in intestinal permeability and a decrease in DAO activity within the intestinal tract. Supplementation with LCs effectively restored colonic DAO activity, resulting in a reduction of intestinal permeability. Prior research has shown that *L. reuteri* LR1 decreased serum levels of DAO and LPS, thereby mitigating the incidence of diarrhea in weaned pigs (Tang et al. 2021). Additionally, dietary supplementation with probiotics significantly enhanced intestinal DAO activity in chicks (Tong et al. 2023). These results align with our findings suggesting that probiotics possess therapeutic potential for the restoration of intestinal epithelial integrity.

The intestinal epithelial barrier, maintained by TJ proteins, plays a crucial role in limiting the translocation of luminal antigens, bacteria, and toxins. Disruption of this barrier leads to increased intestinal permeability, which is closely linked to the onset and progression of inflammatory diseases (Li et al. 2017). Pro‐inflammatory cytokines such as TNF‐α, IL‐1β, and IL‐6 not only serve as biomarkers of inflammation but also directly impair the structural integrity of tight junctions by altering the expression and distribution of key proteins like occludin and claudin‐1. During chemotherapy‐induced mucositis, these elevated cytokines promote immune cell infiltration and activate intracellular signaling pathways that degrade tight junction components, ultimately exacerbating barrier dysfunction and intestinal permeability (Dmytriv et al. 2024; Li et al. 2017; Qiu et al. 2025). This evidence supports the view that inflammatory markers are not only indicators but also active contributors to intestinal barrier disruption, perpetuating a vicious cycle of inflammation and mucosal injury (García Mansilla et al. 2025; Yu et al. 2024). Furthermore, neutrophil infiltration is another key event in the inflammatory response that aggravates intestinal barrier damage. MPO, an enzyme specifically released by activated neutrophils, is widely recognized as a reliable biomarker for assessing the degree of inflammatory cell infiltration and plays a pivotal role in the pathogenesis of 5FU‐induced intestinal mucositis (Wu et al. 2020). Excessive infiltration of neutrophils amplifies tissue injury by releasing reactive oxygen species and proteolytic enzymes, which further disrupt tight junctions and compromise epithelial integrity. During intestinal inflammation, the balance between pro‐inflammatory and anti‐inflammatory cytokines is essential for the maintenance of epithelial barrier function. TNF‐α, primarily produced by macrophages and immune cells, plays a central role in disrupting TJ protein integrity and increasing epithelial permeability. Elevated TNF‐α levels also induce apoptosis of intestinal epithelial cells via caspase‐3 activation, further weakening the barrier and allowing translocation of luminal contents (Gitter et al. 2000; Li et al. 2017). Conversely, IL‐10, predominantly secreted by regulatory T cells (Tregs), exerts potent anti‐inflammatory effects by suppressing pro‐inflammatory cytokine expression, including TNF‐α, and thereby helps preserve tight junction integrity and reduce intestinal permeability (Liu, Liu, et al. 2024).

Several probiotic strains have demonstrated beneficial effects in restoring this immune balance. For example, *C. butyricum* has been reported to stimulate macrophages and enhance IL‐10 secretion, thereby supporting intestinal immune homeostasis (Hayashi et al. 2013). CBM588, a specific strain of *C. butyricum* , has also been shown to inhibit leukocyte recruitment and infiltration into colonic tissues under inflammatory conditions (Hagihara et al. 2020). In addition, oral administration of 10^9^ CFU of *C. butyricum* significantly reduced intestinal permeability and decreased TNF‐α and IL‐6 levels in a burn‐induced intestinal injury model (Zhang et al. 2020). Similarly, *L. reuteri* has been shown to suppress the production of pro‐inflammatory cytokines, highlighting its potential to modulate inflammatory responses (Jiang et al. 2023). Consistent with these observations, our study demonstrated that supplementation with LCs markedly reduced neutrophil infiltration, as reflected by lower MPO activity, moderately suppressed IL‐1β expression, significantly elevated IL‐10 levels, and enhanced the expression of TJ proteins. These combined effects contributed to improved intestinal barrier integrity and attenuation of mucosal inflammation in 5FU‐induced colitis. Altogether, these findings suggest that the protective effect of LCs is mediated not only through the downregulation of pro‐inflammatory cytokines and the upregulation of IL‐10, but also through the reduction of neutrophil infiltration, thereby breaking the cycle of inflammation‐driven barrier dysfunction.

It has been well established that 5FU induces intestinal mucosal injury by activating multiple inflammatory signaling pathways. Specifically, 5FU promotes the phosphorylation of ERK1/2, JNK, p38 MAPK, IκB, and NF‐κB in colonic tissues and upregulates the expression of inducible nitric oxide synthase (iNOS), while also enhancing NF‐κB nuclear translocation in intestinal epithelial cells (Li et al. 2017). The activation of NF‐κB signaling triggers the production of pro‐inflammatory cytokines, including TNF‐α, IL‐1β, and IL‐6. Notably, TNF‐α can further amplify this inflammatory cascade by activating NF‐κB, which in turn exacerbates TJ disruption and intestinal permeability (Monteiro et al. 2024; Wu et al. 2020). The MAPK signaling cascade is another key mediator of inflammatory gene expression, where NF‐κB functions as a downstream effector, integrating upstream signals and regulating the transcription of inflammatory cytokines (Liu, Liu, et al. 2024). Previous studies have demonstrated that probiotics can mitigate intestinal inflammation by inhibiting NF‐κB and MAPK signaling pathways, thereby reducing the release of pro‐inflammatory mediators (Elkholy et al. 2023; Ma et al. 2023; Yu et al. 2024). Consistent with this, our study confirmed that 5FU administration significantly upregulated the mRNA expression of TNF‐α, iNOS, and MAPK in inflamed intestinal mucosa (Li et al. 2017). Probiotic LCs treatment effectively attenuated this response, and although a decreasing trend in NF‐κB expression was observed, this reduction did not reach statistical significance. Several factors might account for this outcome. First, NF‐κB is a rapidly activated and transiently regulated transcription factor, and the time point chosen for tissue collection may not have captured the peak of its activation, leading to an underestimation of its modulation by probiotics. Second, the probiotic combination used in this study may exert strain‐specific protective effects that involve alternative mechanisms beyond the NF‐κB and MAPK pathways, as suggested by other studies (Batista et al. 2022; Hagihara et al. 2020; Yue et al. 2025). Third, the inherent individual variability in the host response to 5FU‐induced intestinal injury may have introduced biological fluctuations in NF‐κB expression, which could have further influenced the statistical results. Taken together, while our study confirms the beneficial effects of *L. reuteri* and *C. butyricum* in alleviating 5FU‐induced colitis, the precise mechanisms by which these probiotics exert their protective effects remain complex and multifactorial. However, the pathogenesis of chemotherapy‐induced intestinal mucositis is complex and has not yet been determined (Yu, Zhang, Guo, et al. 2022). Further research is warranted to clarify the precise molecular mechanisms by which probiotics modulate inflammatory signaling and oxidative stress responses during mucosal injury (Hagihara et al. 2020; Jiang et al. 2023).

Chemotherapy‐induced mucositis is strongly associated with intestinal epithelial cell (IEC) apoptosis, primarily due to the cytotoxic effects of 5FU (Xiang et al. 2020). Platelet factor 4 (PF4/CXCL4), a heparin‐binding protein, functions as a chemotactic cytokine that facilitates the recruitment of leukocytes to inflammatory sites. The pro‐inflammatory chemokine CXCL4 and the activation of the p38‐MAPK signaling pathway lead to the upregulation of proapoptotic proteins such as p53 and Bax, caspase activation, and IEC apoptosis (Gao et al. 2014). In parallel, excessive TNF‐α promotes apoptosis and disrupts intestinal barrier function via caspase‐3 activation (Pott et al. 2018). Bcl‐2 functions as an anti‐apoptotic protein, while Bax serves as a proapoptotic counterpart. The balance between the proapoptotic protein Bax and the anti‐apoptotic protein Bcl‐2 is a critical determinant of intestinal epithelial cell survival. Intestinal toxicity‐induced disruption of this balance leads to an increased Bax/Bcl‐2 ratio, promoting cytochrome c release from mitochondria and sequential activation of caspase‐9 and caspase‐3, thereby initiating apoptosis (Alkushi et al. 2022). Caspase‐3, a key executioner of apoptosis, has been implicated in TNF‐α‐induced intestinal barrier dysfunction by promoting epithelial cell apoptosis (Geng et al. 2024). Probiotic intervention has been shown to modulate this apoptotic process. *L. reuteri* downregulates caspase‐3 expression while enhancing anti‐apoptotic Bcl‐xl, thereby preserving barrier integrity (Zhou et al. 2022). Similarly, administration of *C. butyricum* significantly attenuated both epithelial apoptosis and inflammation in experimental colitis models (Wu, Zhou, et al. 2022). In the colitis model, combined LCs supplementation significantly suppressed the expression of TNF‐α, CXCL4, and caspase‐3, while enhancing occludin expression, thereby protecting against 5FU‐induced colonic apoptosis and barrier disruption. Consistently, our unpublished data further demonstrated that LCs administration markedly downregulated proapoptotic markers Bax and caspase‐3, while restoring the expression of the anti‐apoptotic protein Bcl‐2 in a 5FU‐induced intestinal mucositis model. Moreover, LCs‐derived SCFAs were shown to enhance the activities of antioxidant enzymes, including CAT and GPx, thereby mitigating oxidative stress‐induced apoptosis (Ferrer et al. 2024). Collectively, these findings suggest that the anti‐apoptotic effects of LCs are mediated through both immunomodulatory and antioxidative mechanisms.

Diarrhea is a common adverse effect associated with 5FU‐induced intestinal mucositis. Aquaporins (AQPs), a family of membrane channel proteins responsible for regulating water and small solute transport, play an essential role in maintaining intestinal fluid homeostasis. Dysregulated expression of AQPs can impair water absorption and secretion, thereby exacerbating the pathological diarrhea characteristic of 5FU‐induced intestinal mucositis (Wu et al. 2021). Previous studies have demonstrated that increased intestinal permeability is associated with the altered expression of AQPs. Specifically, 5FU treatment has been reported to upregulate colonic protein levels of AQP3, AQP7, and AQP11, ultimately disrupting water transport and contributing to the onset of diarrhea (Gan et al. 2020). In addition, vasoactive intestinal polypeptide (VIP), one of the most abundant neuropeptides in the gastrointestinal tract, has also been implicated in this process. Dysregulation of water transport, alongside the overexpression of VIP, cyclic adenosine monophosphate (cAMP), and protein kinase A (PKA), has been observed in 5FU‐induced intestinal mucositis, suggesting that the VIP‐cAMP‐PKA signaling axis may further contribute to impaired intestinal water absorption (Wu et al. 2021). Probiotics have shown potential in alleviating diarrhea, likely by modulating AQP expression. A previous study reported that the anti‐diarrheal effects of probiotics are associated with the restoration of water transport and absorption in the colon, as well as the normalization of AQP3 and AQP8 expression (Zhang et al. 2018). Conversely, other research has demonstrated that probiotic supplementation can increase AQP3 expression in models of constipation, highlighting that AQP regulation may exhibit context‐dependent, bidirectional effects depending on the nature of the gastrointestinal disorder (Deng et al. 2018). Consistent with these findings, the present study showed that 5FU administration significantly increased the mRNA expression levels of AQP7, VIP, and PKA, whereas treatment with LCs attenuated this upregulation, suggesting a protective role of LCs in restoring intestinal water transport balance.

According to a study by L. Wu et al. (Wu et al. 2023), 5FU has been shown to induce intestinal inflammation while simultaneously reducing the production of SCFAs. This observation further emphasizes the pivotal role of SCFAs in maintaining intestinal health and highlights their potential involvement as risk‐modulating factors in the pathogenesis of colitis (Xu et al. 2022). Emerging evidence suggests that probiotic supplementation may alleviate intestinal inflammation, in part by promoting SCFA production, thereby preserving the integrity and function of the intestinal mucosal barrier (Shen et al. 2021; Wu, Jha, et al. 2022). SCFAs, as key microbial metabolites, have been reported to exhibit strong negative correlations with pro‐inflammatory cytokines such as TNF‐α, IL‐1β, IFN‐γ, IL‐6, and MPO, while showing a positive correlation with the anti‐inflammatory cytokine IL‐10 (Wu, Jha, et al. 2022). These findings underscore the essential role of SCFAs in supporting both the structural integrity and immune balance of the intestinal epithelium. Furthermore, butyrate, a major SCFA, serves as an important energy substrate for colonocytes. Interestingly, LCs enhanced butyrate production in cisplatin‐induced nephrotoxicity (Hsiao et al. 2021), but only increased total SCFA levels in 5FU‐induced mucosal injury. This discrepancy is likely attributable to variations in the duration of probiotic supplementation and pharmacological exposure. These results highlight the significance of strain selection, dosing strategy, and treatment timing, further reinforcing the translational potential of our findings.

Growing evidence suggests that multistrain probiotic combinations offer superior therapeutic efficacy to single‐strain probiotics in managing colitis, likely due to their synergistic interactions (Hua et al. 2025; Li et al. 2024). In the present study, coadministration of *C. butyricum* and *L. reuteri* markedly alleviated 5FU‐induced colitis, supporting a cooperative protective effect. The underlying mechanisms of this benefit are believed to arise from the complementary actions of both strains. Both *C. butyricum* and *L. reuteri* produce SCFAs, which enhance intestinal barrier integrity by upregulating TJ proteins, stimulating mucus secretion, and promoting epithelial renewal (Cazzaniga et al. 2024). Additionally, both strains produce bioactive compounds such as surface layer proteins, organic acids, antimicrobial peptides, enzymes, and exopolysaccharides, all contributing to anti‐inflammatory and antioxidant properties (García Mansilla et al. 2025). Specifically, *L. reuteri* enhances epithelial adhesion and immune modulation via structural components such as teichoic acid, lipoteichoic acid, and EPS, and produces metabolites including reuterin, indole derivatives, adenosine, and histamine, all of which exhibit immunoregulatory and anti‐inflammatory activities (Ali et al. 2024; Lee et al. 2025). In parallel, *C. butyricum* promotes the production of lipid‐derived specialized proresolving mediators, such as Protectin D1, which enhances IL‐10 secretion and maintains mucosal homeostasis (Cazzaniga et al. 2024). In addition to the direct interactions between probiotics and the host, emerging evidence indicates that communication is also mediated by bioactive molecules secreted by probiotics. Among these, probiotic‐derived extracellular vesicles (EVs) have attracted growing attention for their role in modulating host physiology. Specifically, *C. butyricum* and *L. reuteri* strains secrete EVs, which have been shown to inhibit inflammatory responses and strengthen intestinal barrier function, thereby alleviating colitis (Ma et al. 2023, 2022; Yue et al. 2025). Beyond their local effects on the gut, both strains have demonstrated clinical relevance. *C. butyricum* has been shown to alleviate chemotherapy‐induced diarrhea and systemic inflammation and even enhance the antitumor efficacy of 5FU (Lo et al. 2023; Tian et al. 2019). The probiotic *L. reuteri* has the potential to reduce both local and systemic inflammation and may alleviate gastrointestinal symptoms (Jiang et al. 2023). Notably, we observed that the combination of *C. butyricum* and *L. reuteri* alleviates 5FU‐induced diarrhea and mucositis while also reducing cisplatin‐induced nephrotoxicity and gastrointestinal toxicity, suggesting broad‐spectrum protective potential (Hsiao et al. 2021). These findings highlight the therapeutic promise of probiotic‐based interventions in mitigating chemotherapy‐associated complications.

Our study acknowledges several limitations that offer opportunities for future exploration. First, while 5FU is widely used in preclinical models (Monteiro et al. 2024; Qiu et al. 2025; Shen et al. 2021; Zheng et al. 2023), clinical chemotherapy regimens such as FOLFOXIRI often involve multiple agents, including irinotecan and oxaliplatin (Chai et al. 2024). Future studies should therefore investigate the effects of LCs in combination with chemotherapy models to better simulate clinical settings and evaluate their potential as an adjunctive therapy. Second, the underlying mechanisms by which LCs alleviate chemotherapy‐induced intestinal injury remain incompletely understood. Future research should explore the involvement of molecular pathways such as NF‐κB/MAPK and the regulation of inflammatory cytokines via the TLR4/MyD88/NF‐κB signaling cascade. In addition, untargeted metabolomics analyses of serum, gut microbiota, and colonic tissue may provide deeper insights into the metabolic interactions underlying LCs' therapeutic effects (Liu, Qiu, et al. 2024; Yu, Zhang, Zhao, et al. 2022). Finally, the present study did not assess LCs' effects in a tumor‐bearing model. Previous clinical findings have shown that probiotics can alleviate chemotherapy‐induced gastrointestinal complications without compromising anti‐tumor efficacy in colorectal cancer patients (Huang et al. 2023). Future studies should validate the protective and synergistic roles of LCs in tumor models under active chemotherapy.

## 5 Conclusions

LCs pretreatment effectively alleviated 5FU‐induced diarrhea, likely through the suppression of colonic inflammatory responses, the upregulation of tight junction protein expression, and the reduction of intestinal permeability by decreasing adhesion molecule expression. Moreover, LCs inhibited the AQP‐VIP‐PKA signaling pathway and modulated the CXCL4‐mediated MAPK/caspase‐3 axis, thereby attenuating apoptosis. These findings suggest that LCs supplementation may serve as an effective strategy for the prevention of 5FU‐induced diarrhea.

## Author Contributions

**Sheau‐Chung Tang:** conceptualization (equal), methodology (equal), resources (equal), writing – original draft (equal). **Wen‐Chen Chuang:** conceptualization (equal), investigation (equal), methodology (equal), resources (equal), writing – original draft (equal). **Jiuan‐Miaw Liao:** methodology (equal), resources (equal). **Yi‐Hsuan Tsai:** data curation (equal), investigation (equal), methodology (equal). **Liang‐Chuan Chen:** data curation (equal), formal analysis (equal), investigation (equal). **Jiunn‐Wang Liao:** investigation (equal), methodology (equal). **Jiunn‐Liang Ko:** conceptualization (equal), methodology (equal), writing – review and editing (equal). **Chu‐Chyn Ou:** conceptualization (equal), funding acquisition (equal), methodology (equal), project administration (equal), supervision (equal), writing – review and editing (equal).

## Conflicts of Interest

The authors declare no conflicts of interest.

## Data Availability

The data sets used and/or analyzed during the current study are available from the corresponding author on reasonable request.

## References

[fsn370318-bib-0001] Ali, M. S. , E. B. Lee , S. K. Lim , K. Suk , and S. C. Park . 2023. “Isolation and Identification of Limosilactobacillus Reuteri PSC102 and Evaluation of Its Potential Probiotic, Antioxidant, and Antibacterial Properties.” Antioxidants (Basel) 12, no. 2: 238. 10.3390/antiox12020238. 36829797 PMC9952246

[fsn370318-bib-0002] Ali, M. S. , E. B. Lee , Y. Quah , et al. 2024. “Modulating Effects of Heat‐Killed and Live Limosilactobacillus Reuteri PSC102 on the Immune Response and Gut Microbiota of Cyclophosphamide‐Treated Rats.” Veterinary Quarterly 44, no. 1: 1–18. 10.1080/01652176.2024.2344765. PMC11060015 38682319

[fsn370318-bib-0003] Alkushi, A. G. , A. Abdelfattah‐Hassan , H. Eldoumani , et al. 2022. “Probiotics‐Loaded Nanoparticles Attenuated Colon Inflammation, Oxidative Stress, and Apoptosis in Colitis.” Scientific Reports 12, no. 1: 5116. 10.1038/s41598-022-08915-5. 35332200 PMC8948303

[fsn370318-bib-0004] Ariyoshi, T. , M. Hagihara , S. Tomono , et al. 2021. “ *Clostridium Butyricum* MIYAIRI 588 Modifies Bacterial Composition Under Antibiotic‐Induced Dysbiosis for the Activation of Interactions via Lipid Metabolism Between the Gut Microbiome and the Host.” Biomedicine 9, no. 8: 1065. 10.3390/biomedicines9081065. PMC8391242 34440269

[fsn370318-bib-0005] Batista, V. L. , L. C. L. De Jesus , L. M. Tavares , et al. 2022. “Paraprobiotics and Postbiotics of *Lactobacillus delbrueckii* CIDCA 133 Mitigate 5‐FU‐Induced Intestinal Inflammation.” Microorganisms 10, no. 7: 1418. 10.3390/microorganisms10071418. 35889136 PMC9324481

[fsn370318-bib-0006] Cazzaniga, M. , M. Cardinali , F. Di Pierro , et al. 2024. “The Potential Role of Probiotics, Especially Butyrate Producers, in the Management of Gastrointestinal Mucositis Induced by Oncologic Chemo‐Radiotherapy.” International Journal of Molecular Sciences 25, no. 4: 2306. 10.3390/ijms25042306. 38396981 PMC10889689

[fsn370318-bib-0007] Chai, Y. , J. L. Liu , S. Zhang , et al. 2024. “The Effective Combination Therapies With Irinotecan for Colorectal Cancer.” Frontiers in Pharmacology 15: 1356708. 10.3389/fphar.2024.1356708. 38375031 PMC10875015

[fsn370318-bib-0008] Chen, K. J. , Y. L. Chen , S. H. Ueng , T. L. Hwang , L. M. Kuo , and P. W. Hsieh . 2021. “Neutrophil Elastase Inhibitor (MPH‐966) Improves Intestinal Mucosal Damage and Gut Microbiota in a Mouse Model of 5‐Fluorouracil‐Induced Intestinal Mucositis.” Biomedicine & Pharmacotherapy 134: 111152. 10.1016/j.biopha.2020.111152. 33373916

[fsn370318-bib-0009] Chen, S. , K. Qian , G. Zhang , and M. Zhang . 2022. “Akkermansia Muciniphila and Its Outer Membrane Protein Amuc_1100 Prophylactically Attenuate 5‐Fluorouracil‐Induced Intestinal Mucositis.” Biochemical and Biophysical Research Communications 614: 34–40. 10.1016/j.bbrc.2022.04.135. 35567942

[fsn370318-bib-0010] Chrysostomou, D. , L. A. Roberts , J. R. Marchesi , and J. M. Kinross . 2023. “Gut Microbiota Modulation of Efficacy and Toxicity of Cancer Chemotherapy and Immunotherapy.” Gastroenterology 164, no. 2: 198–213. 10.1053/j.gastro.2022.10.018. 36309208

[fsn370318-bib-0011] Chu, Z. X. , H. Q. Chen , Y. L. Ma , et al. 2010. “ *Lactobacillus plantarum* Prevents the Upregulation of Adhesion Molecule Expression in an Experimental Colitis Model.” Digestive Diseases and Sciences 55, no. 9: 2505–2513. 10.1007/s10620-009-1063-2. 19960256

[fsn370318-bib-0012] Deng, Y. , M. Li , L. Mei , et al. 2018. “Manipulation of Intestinal Dysbiosis by a Bacterial Mixture Ameliorates Loperamide‐Induced Constipation in Rats.” Beneficial Microbes 9, no. 3: 453–464. 10.3920/BM2017.0062. 29633634

[fsn370318-bib-0013] Dmytriv, T. R. , K. B. Storey , and V. I. Lushchak . 2024. “Intestinal Barrier Permeability: The Influence of Gut Microbiota, Nutrition, and Exercise.” Frontiers in Physiology 15: 1380713. 10.3389/fphys.2024.1380713. 39040079 PMC11260943

[fsn370318-bib-0014] Elkholy, S. E. , S. A. Maher , N. R. Abd El‐Hamid , et al. 2023. “The Immunomodulatory Effects of Probiotics and Azithromycin in Dextran Sodium Sulfate‐Induced Ulcerative Colitis in Rats via TLR4‐NF‐kappaB and p38‐MAPK Pathway.” Biomedicine & Pharmacotherapy 165: 115005. 10.1016/j.biopha.2023.115005. 37327586

[fsn370318-bib-0015] Feng, T. , and J. Wang . 2020. “Oxidative Stress Tolerance and Antioxidant Capacity of Lactic Acid Bacteria as Probiotic: A Systematic Review.” Gut Microbes 12, no. 1: 1801944. 10.1080/19490976.2020.1801944. 32795116 PMC7524341

[fsn370318-bib-0016] Ferrer, M. , B. Buey , L. Grasa , et al. 2024. “Protective Role of Short‐Chain Fatty Acids on Intestinal Oxidative Stress Induced by TNF‐Alpha.” Cell Stress & Chaperones 29, no. 6: 769–776. 10.1016/j.cstres.2024.11.002. 39547594 PMC11650142

[fsn370318-bib-0017] Gan, Y. , G. Ai , J. Wu , et al. 2020. “Patchouli Oil Ameliorates 5‐Fluorouracil‐Induced Intestinal Mucositis in Rats via Protecting Intestinal Barrier and Regulating Water Transport.” Journal of Ethnopharmacology 250: 112519. 10.1016/j.jep.2019.112519. 31883475

[fsn370318-bib-0018] Gao, J. , J. Gao , L. Qian , et al. 2014. “Activation of p38‐MAPK by CXCL4/CXCR3 Axis Contributes to p53‐Dependent Intestinal Apoptosis Initiated by 5‐Fluorouracil.” Cancer Biology & Therapy 15, no. 8: 982–991. 10.4161/cbt.29114. 24800927 PMC4119083

[fsn370318-bib-0019] García Mansilla, M. J. , M. J. Rodríguez Sojo , A. R. Lista , et al. 2025. “Microbial‐Derived Antioxidants in Intestinal Inflammation: A Systematic Review of Their Therapeutic Potential.” Antioxidants 14, no. 3: 321. 10.3390/antiox14030321. 40227262 PMC11939483

[fsn370318-bib-0020] Geng, Z. , L. Zuo , J. Li , et al. 2024. “Ginkgetin Improved Experimental Colitis by Inhibiting Intestinal Epithelial Cell Apoptosis Through EGFR/PI3K/AKT Signaling.” FASEB Journal 38, no. 14: e23817. 10.1096/fj.202400211RR. 39003633

[fsn370318-bib-0021] Gitter, A. H. , K. Bendfeldt , J. D. Schulzke , and M. Fromm . 2000. “Leaks in the Epithelial Barrier Caused by Spontaneous and TNF‐Alpha‐Induced Single‐Cell Apoptosis.” FASEB Journal 14, no. 12: 1749–1753. 10.1096/fj.99-0898com. 10973924

[fsn370318-bib-0022] Hagihara, M. , Y. Kuroki , T. Ariyoshi , et al. 2020. “ *Clostridium Butyricum* Modulates the Microbiome to Protect Intestinal Barrier Function in Mice With Antibiotic‐Induced Dysbiosis.” IScience 23, no. 1: 100772. 10.1016/j.isci.2019.100772. 31954979 PMC6970176

[fsn370318-bib-0023] Hayashi, A. , T. Sato , N. Kamada , et al. 2013. “A Single Strain of *Clostridium butyricum* Induces Intestinal IL‐10‐Producing Macrophages to Suppress Acute Experimental Colitis in Mice.” Cell Host & Microbe 13, no. 6: 711–722. 10.1016/j.chom.2013.05.013. 23768495

[fsn370318-bib-0024] Hsiao, Y. P. , H. L. Chen , J. N. Tsai , et al. 2021. “Administration of *Lactobacillus Reuteri* Combined With *Clostridium Butyricum* Attenuates Cisplatin‐Induced Renal Damage by Gut Microbiota Reconstitution, Increasing Butyric Acid Production, and Suppressing Renal Inflammation.” Nutrients 13, no. 8: 2792. 10.3390/nu13082792. 34444952 PMC8402234

[fsn370318-bib-0025] Hua, D. , Q. Yang , X. Li , et al. 2025. “The Combination of Clostridium Butyricum and *Akkermansia muciniphila* Mitigates DSS‐Induced Colitis and Attenuates Colitis‐Associated Tumorigenesis by Modulating Gut Microbiota and Reducing CD8(+) T Cells in Mice.” mSystems 10, no. 2: e0156724. 10.1128/msystems.01567-24. 39840995 PMC11834468

[fsn370318-bib-0026] Huang, F. , S. Li , W. Chen , et al. 2023. “Postoperative Probiotics Administration Attenuates Gastrointestinal Complications and Gut Microbiota Dysbiosis Caused by Chemotherapy in Colorectal Cancer Patients.” Nutrients 15, no. 2: 356. 10.3390/nu15020356. 36678227 PMC9861237

[fsn370318-bib-0027] Jalali, S. , N. Mojgani , M. R. Sanjabi , S. Saremnezhad , and S. Haghighat . 2024. “Functional Properties and Safety Traits of L. Rhamnosus and *L. Reuteri* Postbiotic Extracts.” AMB Express 14, no. 1: 114. 10.1186/s13568-024-01768-3. 39384663 PMC11465093

[fsn370318-bib-0028] Jiang, J. , K. Li , Y. Xiao , et al. 2023. “Limosilactobacillus Reuteri Regulating Intestinal Function: A Review.” Fermentation 9, no. 1: 19. 10.3390/fermentation9010019.

[fsn370318-bib-0029] Justino, P. F. C. , A. X. Franco , R. Pontier‐Bres , et al. 2020. “Modulation of 5‐Fluorouracil Activation of Toll‐Like/MyD88/NF‐kappaB/MAPK Pathway by Saccharomyces Boulardii CNCM I‐745 Probiotic.” Cytokine 125: 154791. 10.1016/j.cyto.2019.154791. 31401369

[fsn370318-bib-0030] Kim, S. H. , H. J. Chun , H. S. Choi , et al. 2018. “Ursodeoxycholic Acid Attenuates 5‐Fluorouracil‐Induced Mucositis in a Rat Model.” Oncology Letters 16, no. 2: 2585–2590. 10.3892/ol.2018.8893. 30008943 PMC6036549

[fsn370318-bib-0031] Ksonzekova, P. , P. Bystricky , S. Vlckova , et al. 2016. “Exopolysaccharides of *Lactobacillus reuteri* : Their Influence on Adherence of *E. coli* to Epithelial Cells and Inflammatory Response.” Carbohydrate Polymers 141: 10–19. 10.1016/j.carbpol.2015.12.037. 26876991

[fsn370318-bib-0032] Kuo, C. H. , S. H. Lee , K. M. Chen , C. K. Lii , and C. T. Liu . 2011. “Effect of Garlic Oil on Neutrophil Infiltration in the Small Intestine of Endotoxin‐Injected Rats and Its Association With Levels of Soluble and Cellular Adhesion Molecules.” Journal of Agricultural and Food Chemistry 59, no. 14: 7717–7725. 10.1021/jf201185v. 21688797

[fsn370318-bib-0033] Lee, A. H. , D. M. Rodriguez Jimenez , and M. Meisel . 2025. “Limosilactobacillus Reuteri—A Probiotic Gut Commensal With Contextual Impact on Immunity.” Gut Microbes 17, no. 1: 2451088. 10.1080/19490976.2025.2451088. 39825615 PMC12716054

[fsn370318-bib-0034] Li, H. L. , L. Lu , X. S. Wang , et al. 2017. “Alteration of Gut Microbiota and Inflammatory Cytokine/Chemokine Profiles in 5‐Fluorouracil Induced Intestinal Mucositis.” Frontiers in Cellular and Infection Microbiology 7: 455. 10.3389/fcimb.2017.00455. 29124041 PMC5662589

[fsn370318-bib-0035] Li, W. , Y. Zhang , M. Chen , X. Guo , and Z. Ding . 2024. “The Antioxidant Strain Lactiplantibacillus Plantarum AS21 and *Clostridium butyricum* Ameliorate DSS‐Induced Colitis in Mice by Remodeling the Assembly of Intestinal Microbiota and Improving Gut Functions.” Food & Function 15, no. 4: 2022–2037. 10.1039/d3fo05337g. 38289370

[fsn370318-bib-0036] Liu, Q. , L. Cheng , M. Wang , et al. 2024. “Dietary Sodium Acetate and Sodium Butyrate Improve High‐Carbohydrate Diet Utilization by Regulating Gut Microbiota, Liver Lipid Metabolism, Oxidative Stress, and Inflammation in Largemouth Bass ( *Micropterus salmoides* ).” Journal of Animal Science and Biotechnology 15, no. 1: 50. 10.1186/s40104-024-01009-4. 38566217 PMC10988814

[fsn370318-bib-0037] Liu, X. , X. Qiu , Y. Yang , et al. 2024. “Uncovering the Mechanism of *Clostridium Butyricum* CBX 2021 to Improve Pig Health Based on In Vivo and In Vitro Studies.” Frontiers in Microbiology 15: 1394332. 10.3389/fmicb.2024.1394332. 38946904 PMC11211278

[fsn370318-bib-0038] Liu, Y. , G. Liu , and J. Fang . 2024. “Progress on the Mechanisms of *Lactobacillus Plantarum* to Improve Intestinal Barrier Function in Ulcerative Colitis.” Journal of Nutritional Biochemistry 124: 109505. 10.1016/j.jnutbio.2023.109505. 37890709

[fsn370318-bib-0039] Lo, E. K. K. , H. K. M. Leung , F. Zhang , and H. El‐Nezami . 2023. “Gut Microbiota: Impact on 5‐Fluorouracil Efficacy and Toxicity.” Current Opinion in Toxicology 36: 100423. 10.1016/j.cotox.2023.100423.

[fsn370318-bib-0040] Lopez‐Gomez, L. , A. Alcorta , and R. Abalo . 2023. “Probiotics and Probiotic‐Like Agents Against Chemotherapy‐Induced Intestinal Mucositis: A Narrative Review.” Journal of Personalized Medicine 13, no. 10: 1487. 10.3390/jpm13101487. 37888098 PMC10607965

[fsn370318-bib-0041] Ma, L. , W. Lyu , Y. Song , et al. 2023. “Anti‐Inflammatory Effect of *Clostridium butyricum* ‐Derived Extracellular Vesicles in Ulcerative Colitis: Impact on Host microRNAs Expressions and Gut Microbiome Profiles.” Molecular Nutrition & Food Research 67, no. 13: e2200884. 10.1002/mnfr.202200884. 37183784

[fsn370318-bib-0042] Ma, L. , Q. Shen , W. Lyu , et al. 2022. “Clostridium Butyricum and Its Derived Extracellular Vesicles Modulate Gut Homeostasis and Ameliorate Acute Experimental Colitis.” Microbiology Spectrum 10, no. 4: e0136822. 10.1128/spectrum.01368-22. 35762770 PMC9431305

[fsn370318-bib-0043] Maftei, N. M. , C. R. Raileanu , A. A. Balta , et al. 2024. “The Potential Impact of Probiotics on Human Health: An Update on Their Health‐Promoting Properties.” Microorganisms 12, no. 2: 234. 10.3390/microorganisms12020234. 38399637 PMC10891645

[fsn370318-bib-0044] Monteiro, C. , B. de Cerqueira Fiorio , F. G. O. Silva , et al. 2024. “A Polyphenol‐Rich Acai Seed Extract Protects Against 5‐Fluorouracil‐Induced Intestinal Mucositis in Mice Through the TLR‐4/MyD88/PI3K/mTOR/NF‐kappaBp65 Signaling Pathway.” Nutrition Research 125: 1–15. 10.1016/j.nutres.2024.01.017. 38428258

[fsn370318-bib-0045] Pott, J. , A. M. Kabat , and K. J. Maloy . 2018. “Intestinal Epithelial Cell Autophagy Is Required to Protect Against TNF‐Induced Apoptosis During Chronic Colitis in Mice.” Cell Host & Microbe 23, no. 2: 191–202. 10.1016/j.chom.2017.12.017. 29358084

[fsn370318-bib-0046] Qiu, Y. T. , X. Y. Luo , Y. F. Deng , et al. 2025. “Modified Pulsatilla Decoction Alleviates 5‐Fluorouracil‐Induced Intestinal Mucositis by Modulating the TLR4/MyD88/NF‐kappaB Pathway and Gut Microbiota.” World Journal of Gastroenterology 31, no. 7: 98806. 10.3748/wjg.v31.i7.98806. 39991674 PMC11755253

[fsn370318-bib-0047] Rezazadeh, L. , S. Pourmoradian , H. Tutunchi , N. Farrin , N. Radkhah , and A. Ostadrahimi . 2023. “The Effects of Probiotics on VCAM‐1 and ICAM‐1: A Systematic Review and Meta‐Analysis of Randomized Controlled Trials.” Clinical Nutrition ESPEN 54: 60–67. 10.1016/j.clnesp.2023.01.009. 36963899

[fsn370318-bib-0048] Sang, L. X. , B. Chang , C. Dai , N. Gao , W.‐X. Liu , and M. Jiang . 2013. “Heat‐Killed VSL#3 Ameliorates Dextran Sulfate Sodium (DSS)‐Induced Acute Experimental Colitis in Rats.” International Journal of Molecular Sciences 15, no. 1: 15–28. 10.3390/ijms15010015. 24451125 PMC3907795

[fsn370318-bib-0049] Shen, S. R. , W. J. Chen , H. F. Chu , S. H. Wu , Y. R. Wang , and T. L. Shen . 2021. “Amelioration of 5‐Fluorouracil‐Induced Intestinal Mucositis by *Streptococcus thermophilus* ST4 in a Mouse Model.” PLoS One 16, no. 7: e0253540. 10.1371/journal.pone.0253540. 34310611 PMC8312939

[fsn370318-bib-0050] Shi, P. , T. Zhao , W. Wang , et al. 2022. “Protective Effect of Homogeneous Polysaccharides of Wuguchong (HPW) on Intestinal Mucositis Induced by 5‐Fluorouracil in Mice.” Nutrition & Metabolism (London) 19, no. 1: 36. 10.1186/s12986-022-00669-1. PMC9118848 35585561

[fsn370318-bib-0051] Siritientong, T. , D. Thet , N. Leelakanok , and N. Areepium . 2025. “Oral Probiotic Supplementation to Alleviate Diarrhea Induced by Fluoropyrimidines or Irinotecan‐Based Chemotherapy: A Systematic Review and Meta‐Analysis.” Complementary Therapies in Medicine 89: 103151. 10.1016/j.ctim.2025.103151. 39993479

[fsn370318-bib-0052] Tang, Q. , H. Yi , W. Hong , et al. 2021. “Comparative Effects of *L. Plantarum* CGMCC 1258 and *L. Reuteri* LR1 on Growth Performance, Antioxidant Function, and Intestinal Immunity in Weaned Pigs.” Frontiers in Veterinary Science 8: 728849. 10.3389/fvets.2021.728849. 34859082 PMC8632148

[fsn370318-bib-0053] Tian, Y. , M. Li , W. Song , R. Jiang , and Y. Q. Li . 2019. “Effects of Probiotics on Chemotherapy in Patients With Lung Cancer.” Oncology Letters 17, no. 3: 2836–2848. 10.3892/ol.2019.9906. 30854059 PMC6365978

[fsn370318-bib-0054] Tong, D. Q. , Z. J. Lu , N. Zeng , X. Q. Wang , H. C. Yan , and C. Q. Gao . 2023. “Dietary Supplementation With Probiotics Increases Growth Performance, Improves the Intestinal Mucosal Barrier and Activates the Wnt/Beta‐Catenin Pathway Activity in Chicks.” Journal of the Science of Food and Agriculture 103, no. 9: 4649–4659. 10.1002/jsfa.12562. 36930725

[fsn370318-bib-0055] Wang, W. , B. Cui , Y. Nie , L. Sun , and F. Zhang . 2023. “Radiation Injury and Gut Microbiota‐Based Treatment.” Protein & Cell 15, no. 2: 83–97. 10.1093/procel/pwad044. PMC10833463 37470727

[fsn370318-bib-0056] Wen, J. J. , G. Vyatkina , and N. Garg . 2004. “Oxidative Damage During Chagasic Cardiomyopathy Development: Role of Mitochondrial Oxidant Release and Inefficient Antioxidant Defense.” Free Radical Biology and Medicine 37, no. 11: 1821–1833. 10.1016/j.freeradbiomed.2004.08.018. 15528041

[fsn370318-bib-0057] Wu, C. H. , J. L. Ko , J. M. Liao , et al. 2019. “D‐Methionine Alleviates Cisplatin‐Induced Mucositis by Restoring the Gut Microbiota Structure and Improving Intestinal Inflammation.” Therapeutic Advances in Medical Oncology 11: 1758835918821021. 10.1177/1758835918821021. 30792823 PMC6376546

[fsn370318-bib-0058] Wu, J. , Y. Gan , M. Li , et al. 2020. “Patchouli Alcohol Attenuates 5‐Fluorouracil‐Induced Intestinal Mucositis via TLR2/MyD88/NF‐kB Pathway and Regulation of Microbiota.” Biomedicine & Pharmacotherapy 124: 109883. 10.1016/j.biopha.2020.109883. 32004938

[fsn370318-bib-0059] Wu, J. , Y. Gan , H. Luo , et al. 2021. “Beta‐Patchoulene Ameliorates Water Transport and the Mucus Barrier in 5‐Fluorouracil‐Induced Intestinal Mucositis Rats via the cAMP/PKA/CREB Signaling Pathway.” Frontiers in Pharmacology 12: 689491. 10.3389/fphar.2021.689491. 34512326 PMC8424048

[fsn370318-bib-0060] Wu, J. , B. Zhou , X. Pang , et al. 2022. “ *Clostridium butyricum* , A Butyrate‐Producing Potential Probiotic, Alleviates Experimental Colitis Through Epidermal Growth Factor Receptor Activation.” Food & Function 13, no. 13: 7046–7061. 10.1039/d2fo00478j. 35678197

[fsn370318-bib-0061] Wu, L. , Y. Xi , M. Yan , et al. 2023. “Berberine‐Based Carbon Quantum Dots Improve Intestinal Barrier Injury and Alleviate Oxidative Stress in C57BL/6 Mice With 5‐Fluorouracil‐Induced Intestinal Mucositis by Enhancing Gut‐Derived Short‐Chain Fatty Acids Contents.” Molecules 28, no. 5: 2148. 10.3390/molecules28052148. 36903391 PMC10004514

[fsn370318-bib-0062] Wu, L. , X. Xie , Y. Li , et al. 2022. “Gut Microbiota as an Antioxidant System in Centenarians Associated With High Antioxidant Activities of Gut‐Resident Lactobacillus.” NPJ Biofilms and Microbiomes 8, no. 1: 102. 10.1038/s41522-022-00366-0. 36564415 PMC9789086

[fsn370318-bib-0063] Wu, Y. , R. Jha , A. Li , et al. 2022. “Probiotics ( *Lactobacillus plantarum* HNU082) Supplementation Relieves Ulcerative Colitis by Affecting Intestinal Barrier Functions, Immunity‐Related Gene Expression, Gut Microbiota, and Metabolic Pathways in Mice.” Microbiology Spectrum 10, no. 6: e0165122. 10.1128/spectrum.01651-22. 36321893 PMC9769980

[fsn370318-bib-0064] Xiang, D. C. , J. Y. Yang , Y. J. Xu , et al. 2020. “Protective Effect of Andrographolide on 5‐Fu Induced Intestinal Mucositis by Regulating p38 MAPK Signaling Pathway.” Life Sciences 252: 117612. 10.1016/j.lfs.2020.117612. 32247004

[fsn370318-bib-0065] Xu, H. M. , H. L. Zhao , G. J. Guo , et al. 2022. “Characterization of Short‐Chain Fatty Acids in Patients With Ulcerative Colitis: A Meta‐Analysis.” BMC Gastroenterology 22, no. 1: 117. 10.1186/s12876-022-02191-3. 35272614 PMC8908609

[fsn370318-bib-0066] Yeung, C. Y. , J. C. Chiau , M. L. Cheng , et al. 2021. “Immune Modulation Effects of *Lactobacillus Casei* Variety Rhamnosus on Enterocytes and Intestinal Stem Cells in a 5‐FU‐Induced Mucositis Mouse Model.” Gastroenterology Research and Practice 2021: 1. 10.1155/2021/3068393. PMC7850847 33564301

[fsn370318-bib-0067] Yong, Y. , J. Li , D. Gong , et al. 2021. “ERK1/2 Mitogen‐Activated Protein Kinase Mediates Downregulation of Intestinal Tight Junction Proteins in Heat Stress‐Induced IBD Model in Pig.” Journal of Thermal Biology 101: 103103. 10.1016/j.jtherbio.2021.103103. 34879918

[fsn370318-bib-0068] Yu, Q. Q. , H. Zhang , Y. Guo , B. Han , and P. Jiang . 2022. “The Intestinal Redox System and Its Significance in Chemotherapy‐Induced Intestinal Mucositis.” Oxidative Medicine and Cellular Longevity 2022: 7255497. 10.1155/2022/7255497. 35585883 PMC9110227

[fsn370318-bib-0069] Yu, Q. Q. , H. Zhang , S. Zhao , et al. 2022. “Systematic Evaluation of Irinotecan‐Induced Intestinal Mucositis Based on Metabolomics Analysis.” Frontiers in Pharmacology 13: 958882. 10.3389/fphar.2022.958882. 36188576 PMC9520243

[fsn370318-bib-0070] Yu, S. , J. Xie , Q. Guo , et al. 2024. “ *Clostridium Butyricum* Isolated From Giant Panda Can Attenuate Dextran Sodium Sulfate‐Induced Colitis in Mice.” Frontiers in Microbiology 15: 1361945. 10.3389/fmicb.2024.1361945. 38646621 PMC11027743

[fsn370318-bib-0071] Yue, N. , H. Zhao , P. Hu , et al. 2025. “Real‐World of Limosilactobacillus Reuteri in Mitigation of Acute Experimental Colitis.” Journal of Nanobiotechnology 23, no. 1: 65. 10.1186/s12951-025-03158-8. 39891249 PMC11783912

[fsn370318-bib-0072] Zhang, D. , C. Zhu , Z. Fang , et al. 2020. “Remodeling Gut Microbiota by *Clostridium butyricum* (C.Butyricum) Attenuates Intestinal Injury in Burned Mice.” Burns 46, no. 6: 1373–1380. 10.1016/j.burns.2020.01.007. 32014349

[fsn370318-bib-0073] Zhang, W. , B. Zhu , J. Xu , et al. 2018. “ *Bacteroides Fragilis* Protects Against Antibiotic‐Associated Diarrhea in Rats by Modulating Intestinal Defenses.” Frontiers in Immunology 9: 1040. 10.3389/fimmu.2018.01040. 29868005 PMC5954023

[fsn370318-bib-0074] Zheng, X. , L. Mai , Y. Xu , et al. 2023. “Brucea Javanica Oil Alleviates Intestinal Mucosal Injury Induced by Chemotherapeutic Agent 5‐Fluorouracil in Mice.” Frontiers in Pharmacology 14: 1136076. 10.3389/fphar.2023.1136076. 36895947 PMC9990700

[fsn370318-bib-0075] Zhou, Q. , F. Wu , S. Chen , et al. 2022. “ *Lactobacillus Reuteri* Improves Function of the Intestinal Barrier in Rats With Acute Liver Failure Through Nrf‐2/HO‐1 Pathway.” Nutrition 99‐100: 111673. 10.1016/j.nut.2022.111673. 35567844

[fsn370318-bib-0076] Zhou, T. , S. Qiu , L. Zhang , et al. 2024. “Supplementation of *Clostridium butyricum* Alleviates Vascular Inflammation in Diabetic Mice.” Diabetes & Metabolism Journal 48, no. 3: 390–404. 10.4093/dmj.2023.0109. 38310882 PMC11140397

